# Issues of Using Benzyl Ether in Nanomaterials’ Synthesis: Insights for a Standardized Synthesis of FeWO_*x*_ Nanocrystals and Their Use as Photocatalysts

**DOI:** 10.1021/acsomega.5c07938

**Published:** 2025-09-30

**Authors:** Raúl Boix, M. Pilar Lobera, María Bernechea

**Affiliations:** † Instituto de Nanociencia y Materiales de Aragón (INMA), CSIC-Universidad de Zaragoza, Department of Chemical and Environmental Engineering, C/Mariano Esquillor s/n, Zaragoza 50018, Spain; ‡ Centro de Investigación Biomédica en Red de Bioingeniería, Biomateriales y Nanomedicina, Instituto de Salud Carlos III, C/Mariano Esquillor s/n, Zaragoza 50018, Spain; § ARAID, Government of Aragon, Av. Ranillas, 1-D, Zaragoza 50018, Spain

## Abstract

The synthesis of nonstoichiometric FeWO_*x*_ nanocrystals via thermal decomposition in benzyl ether has been systematically optimized, addressing reproducibility issues typically associated with this solvent. A key finding of this work is the identification of benzoic acid, a benzyl ether oxidation byproduct, as a necessary ligand for stabilizing tungsten intermediates and enabling consistent FeWO_*x*_ formation. The optimized protocol allows fine-tuning of the Fe/W atomic ratio, leading to a series of materials with tailored stoichiometry, surface properties, and electronic structure. Fe1.5WO_*x*_, Fe1.0WO*_x_,* and Fe0.1WO_*x*_ have been selected as representative examples of materials with Fe excess, slight Fe excess, and an Fe/W ratio close to 1. Among them, Fe0.1WO_*x*_ exhibited the best photocatalytic performance in the degradation of rifampicin under simulated solar irradiation, achieving a 67% degradation within 150 min and displaying a kinetic rate constant (0.0076 min^–1^) three times higher than the other compositions. This superior activity is attributed to its reduced band gap (1.45 eV) and favorable band-edge positions. Scavenger experiments confirmed that holes and hydroxyl radicals (^•^OH) are the main reactive species involved in the degradation process. These findings provide key insights for designing reproducible benzyl ether-based syntheses and demonstrate the potential of FeWO_*x*_ nanomaterials for photocatalytic water treatment applications.

## Graphical abstract

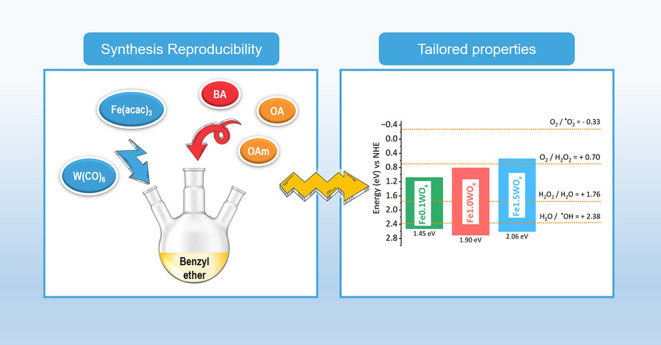

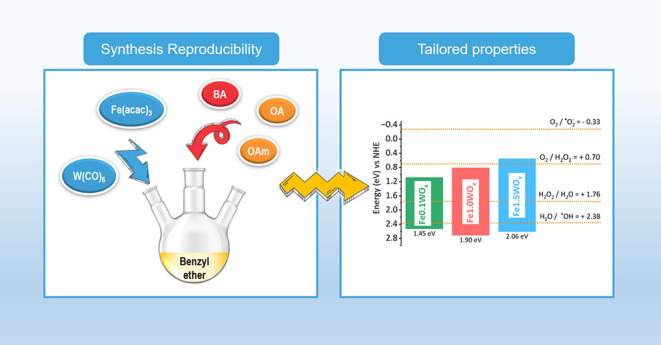

## 1 Introduction

FeWO_4_ oxides are commonly synthesized using hydrothermal methods and employed in various fields such as catalysis^1–4^ and energy storage.^5–8^ These oxides have shown significant potential due to their unique electronic and optical properties,^1,9–12^ presenting band gaps around 1.8–3.0 eV^11,13–16^ (i.e., in the visible light range). A less explored synthetic method for obtaining these materials is the thermal decomposition of metal precursors in high boiling point organic solvents.^17,18^ In this work, FeWO_*x*_ has been synthesized following this approach, according to previously reported procedures that employ benzyl ether as the solvent.^17,18^ However, in some cases, the use of benzyl ether in synthesis is associated with a lack of reproducibility.^19–22^ Qiao et al.^19^ reported that the oxidation of benzyl ether, a solvent typically used for the synthesis of magnetite nanocrystals, plays a key role in the reproducibility of the synthesis. Avasthi et al.^20^ revisited the most commonly used method for producing highly reproducible and scalable iron oxide nanoparticles. They compared different solvents and found that using diphenyl ether as a solvent improved reproducibility, scalability, and polydispersity as compared to benzyl ether. Ivanova et al.^21^ studied the synthesis of magnetic M-ferrite (Mn, Co, and Zn) nanoparticles by thermal decomposition of acetylacetonates. They showed that the use of benzyl ether leads to higher variability in the M:Fe ratio, crystallite size, and nanoparticle size as compared to the syntheses in benzyl alcohol, and they attribute this variability to the generation of volatile products such as benzyl aldehyde or benzyl benzoate from benzyl ether. Hu et al.^22^ analyzed the influence of the solvent in the synthesis of Mn_0.6_Zn_0.4_Fe_2_O nanoparticles by thermal decomposition. Nanoparticles prepared with dibenzyl ether as the solvent showed a higher crystallinity as compared to those synthesized with 1-octadecene and hexadecylamine as solvents. This was explained by the reducing ability of benzanldehyde, generated from the oxidation of dibenzyl ether at high temperatures. Gilbert and Gajewski^23^ were the first to report that benzyl ether leads to the formation of benzene and benzaldehyde at high temperatures via a free-radical chain mechanism. This observation has prompted some recent studies such as those by Mekseriwattana et al.^24^ and Gavilán et al.,^25^ who synthesized iron oxide nanocubes and ferrite nanoparticles, respectively, in benzyl ether, adding controlled amounts of benzaldehyde to tune particle size. Similarly, in related ferrite syntheses, the inclusion of aromatic molecules like 4-biphenyl carboxylic acid^26^ or other additives such as squalane^27^ has also been reported, as they promote the formation of iron oxide nanoparticles and enhance the reproducibility of the synthesis.

Despite this drawback, thermal decomposition is reported to be a method that allows precise control over the morphology and properties of the resulting nanoparticles, delivering monodisperse (FeWO_*x*_) nanoparticles with a nonfixed stoichiometry and multivalent metal elements (Fe^2+^/Fe^3+^ and W^5+^/W^6+^). This redox flexibility can facilitate cycling between different oxidation states, promoting the generation of ROS through photocatalytic reactions or similar mechanisms, without collapse or modification of the structure.^17,18,28^

FeWO_*x*_ oxides have been used in the photocatalytic degradation of organic pollutants in wastewater.^1,4,9–11,29,30^ However, the use of nonstoichiometric FeWO_*x*_ oxides as photocatalysts has not been reported so far, being their main application in biomedicine for cancer therapeutics due to their ability to photogenerate ROS,^17,18,31,32^ which may be of interest in photocatalytic degradation of organic compounds. In the case of other nonstoichiometric oxides such as WO_*x*_, a higher photocatalytic efficiency has been reported as compared to stoichiometric WO_3_.^33,34^ For all of these reasons, it has been considered interesting to study the use of FeWO_*x*_ oxides in photocatalysis for the removal of pollutants in aqueous media.

In this work, we have optimized the synthesis to obtain FeWO_*x*_ nanoparticles with tailored properties in a controlled manner. We have identified benzoic acid, a benzyl ether oxidation byproduct formed during storage, as a necessary ligand for stabilizing tungsten intermediates and ensuring consistent FeWO_*x*_ formation. After the synthesis optimization, we explored the possibilities of controlling the final composition of the FeWO_*x*_ nanoparticles by varying the initial molar Fe/W ratios. The M/M’ ratio in bimetallic systems can be optimized for better efficiency in catalytic,^28^ photocatalytic,^35,36^ or electrocatalytic^37^ applications. The samples were characterized using XRD, FTIR, Raman, TGA, AES, BET, XPS, SEM, TEM, EDX, UV–vis, and cyclic voltammetry, allowing us to determine the different Fe/W ratios and oxygen vacancies. The photocatalytic properties of the prepared FeWO_*x*_ nanoparticles were investigated for the removal of rifampicin antibiotic in aqueous media. In addition, the main reactive species that degrade rifampicin have been studied through inhibition studies.

## 2 Experimental Section

### 2.1 Chemicals and Reagents

Tungsten hexacarbonyl (W­(CO)_6_), oleylamine (approximately C18-content 80–90%) (OAm), and oleic acid ≥ 95% (OA) were obtained from Fisher Scientific. Iron­(III) acetylacetonate (Fe­(acac)_3_), benzyl ether 98% (BE), benzoic acid (BA), benzyl benzoate (BB), benzaldehyde (BZ), 1,2-dodecanediol (90%), ferrocene (Fc), tetrabutylammonium hexafluorophosphate (TBAPF_6_), isopropanol ≥99.5% sodium nitrate (NaNO_3_), and l-ascorbic acid were purchased from Sigma-Aldrich. Sodium chloride (NaCl) was obtained from Panreac.

### 2.2 Synthesis of FeWO_*x*_ Nanoparticles

FeWO_*x*_ nanoparticles were obtained using a previously reported thermal decomposition method in an organic phase with some modifications.^17,18^ In the original reports, 20 mL of benzyl ether, 1.5 g of 1,2-dodecanediol, and 1 mmol of W­(CO)_6_ were added to a three-neck flask. Subsequently, a reflux system was connected and the reaction medium was protected from air with a N_2_ stream. The system was heated under N_2_, and when the temperature reached 120 °C, 2.5 mmol of oleylamine (OAm) and 3 mmol of oleic acid (OA) were injected into the flask. When the mixture reached 260 °C, 1 mmol of Fe precursor (Fe­(acac)_3_) was added, allowing the mixture to react for 10 min at that temperature.

However, several modifications were introduced to the reaction related to (1) the temperature of oleylamine (OAm) and oleic acid (OA) addition; (2) the temperature of Fe precursor incorporation; and (3) the addition of benzoic acid (BA), benzyl benzoate (BB), or benzaldehyde (BZ), depending on the specific procedure, as detailed in Table 1.

**Table 1:** Summary of Synthesized Materials and Reagent Quantities Used per 1 mmol of the W­(CO)_6_ Precursor

Material	mmol of Fe(acac)_3_	Oleic acid (OA)	Benzoic acid (BA)	Benzaldehyde (BZ)	Benzyl benzoate (BB)
FeWO_OA18	1	18	-	-	-
FeWO_OA9	1	9	-	-	-
FeWO_OA3	1	3	-	-	-
FeWO_OA3_BA18	1	3	18	-	-
FeWO_OA3_BA9	1	3	9	-	-
FeWO_OA3_BA3	1	3	3	-	-
FeWO_OL3_BB18	1	3	-	-	18
FeWO_OL3_BZ18	1	3	-	18	-
FeWO_BA18	1	-	18	-	-
FeWO_BA9	1	-	9	-	-
FeWO_BA3	1	-	3	-	-
Fe0.1WO_x_	0.1	3	18	-	-
Fe0.3WO_x_	0.3	3	18	-	-
Fe0.5WO_x_	0.5	3	18	-	-
Fe1.0WO_x_	1	3	18	-	-
Fe1.5WO_x_	1.5	3	18	-	-

In the optimized synthesis, all chemicals (benzyl ether, 1,2-dodecanediol, W­(CO)_6_, oleylamine, oleic acid, and benzoic acid) except Fe­(acac)_3_ were added at room temperature to 20 mL of degassed benzyl ether for 20 min, before connecting the reflux system and the N_2_ stream. In some experiments, benzoic acid was not added or was replaced by benzyl benzoate (BB) or benzaldehyde (BZ), and in one experiment, benzyl ether was replaced by octadecene. The iron precursor was added at 170 °C without stopping the heating, and the mixture was allowed to react for 10 min at 260 °C. In our work, different amounts of Fe­(acac)_3_ powder were incorporated (0.1, 0.3, 0.5, 1, or 1.5 mmol), as detailed in Table 1. After 10 min of reaction at 260 °C, the flask was cooled to room temperature using an ice bath, and an excess of ethanol was used to precipitate the FeWO_*x*_ nanoparticles. The product was purified following several cycles of dispersion-precipitation with 6 mL cyclohexane/3 mL ethanol (13000 rpm, 5 min). Finally, the solid was dried overnight in an oven at 37 °C.

The different materials are formulated as follows: FeWO_X#, where *X* indicates the ligand added and # is the amount in mmol (Table 1).

### 2.3 Characterization

The X-ray diffraction (XRD) patterns were obtained with PANalytical Empyrean equipment in the Bragg–Brentano configuration equipped with a (002) Ge monochromator using the CuKα1 line at 1.5405 Å. Sample patterns were compared with reference standards from the COD database.^38,39^ The size of the crystalline phase of the materials was determined by applying the Scherrer equation as follows:^40–42^

1 $$D=\frac{K\lambda }{\beta \cos\Theta }$$

where *D* is the size of the crystalline phase, *K* is a form constant (∼0.9), λ is the wavelength of the X-ray used, β is the half-height width (fwhm) of the diffraction peak in radians, and Θ is the diffraction angle.

The quantity of Fe and W in FeWO_*x*_ samples was evaluated using a Microwave Plasma-Atomic Emission Spectrometer (4100 MP-AES, Agilent) and Inductively Coupled Plasma-Atomic Emission Spectroscopy (ICP-AES, iCAP PRO, Thermo Scientific). The MP-AES instrument was calibrated with concentration standards 1, 2, 4, 6, 9, and 12 ppm of each metal using a 5:1 (v/v) water/aqua regia mixture as the solvent, and the ICP-AES instrument was calibrated in the same way with calibration standards 0.05, 0.1, 0.5, 1, 5, 10, and 50 ppm. The emission wavelengths selected were 259.940 and 207.911 nm for Fe and W, respectively. To measure each sample in the instruments, 5 mg were digested with 25 mL of aqua regia at 200 °C using a microwave. Then, 1 mL of the digested sample was diluted in 9 mL of a mixture 5:1 (v/v) water/aqua regia.

X-ray photoelectron spectroscopy (XPS) spectra were obtained with the aid of an AXIS Supra (Kratos Tech., Manchester, UK) using a monochromatic Al–Kα source (1486.6 eV) run at 15 kV and 15 mA. High-definition XPS spectra of the elements Fe, W, C, and O were obtained. All spectra were analyzed with the CasaXPS software and calibrated by assigning the C 1s binding energy signal a value of 284.8 eV.

Transmission Electron Microscopy (TEM) and Energy-Dispersive X-ray Spectroscopy (EDX) were made with a Tecnai F30 (FEI) microscope in scanning TEM-EDX mode, operated at 300 kV. Energy-Dispersive X-ray Spectroscopy (EDX) was recorded using a Scanning Electron Microscopy (SEM) INSPECT 50 microscope (FEI, Thermo Fisher, Brno, Czech Republic) in scanning SEM-EDX mode, operated at 5 kV.

FTIR spectra were obtained using a Bruker VERTEX 70 FTIR spectrometer (Bruker, Billerica, MA, USA) equipped with a Golden Gate diamond ATR accessory. Each spectrum was analyzed with a resolution of 40 cm^–1^ and 100 scans.

Raman measurements were carried out using a WITec Alpha 300 confocal benchtop (spectral resolution: 2 cm^–1^), coupled to a 532 nm laser. All measurements were recorded with a 0.5 mW laser power.

UV–vis spectra of liquids were recorded using a JASCO V-670 UV–vis spectrophotometer (JASCO, Tokyo, Japan). UV–vis diffuse reflectance measurements (1300–300 nm) were conducted on a Shimadzu UV–vis 2600 spectrophotometer with an ISR-2600Plus integrating sphere. The band gaps were estimated using Tauc plots assuming direct band gap transitions, representing (R*hν)^2^ versus hν, and extrapolating a straight line to the *x*-axis.

Thermogravimetric analyses (TGAs) were performed with TGA Discovery Q5000 equipment. The measurements were made under 60 mL/min air or N_2_ flow at a heating ramp of 5 °C/min from 40 to 600 °C.

The porosity of the materials was evaluated with N_2_ adsorption at 77 K in a Micromeritics TriStar 3000. The solids were previously degassed at 473 K for 10 h. The specific surface area was calculated from the adsorption isotherm using the Brunauer–Emmett–Teller (BET) method in the range of relative pressure (*p*/*p* ^0^) 0.05–0.4. MicroActive software was used to process these data.

To measure the zeta potential of the materials, colloidal suspensions of the materials were prepared in an aqueous solution of 1 mM KCl at pH 7.5. The results were obtained at 25 °C from 5 measurements of 30 cycles each. The measurements were made with a Brookhaven 90 Plus instrument using ZetaPals software.

### 2.4 Energy Positions of Conduction and Valence Bands

The conduction band (CB) and valence band (VB) energy positions of a semiconductor material can be predicted using the following empirical equations:^43–45^

2 $$E_{VB}=X-E_{e}+0.5E_{g}$$

3 $$E_{CB}=E_{VB}-E_{g}$$

where *X* is the electronegativity of the semiconductor, calculated as the geometric mean of the electronegativity of the constituent atoms; *E*_e_ is the energy of free electrons in the normal hydrogen electrode scale (4.5 eV); and *E*_g_ is the band gap energy of the semiconductor.

To calculate the *X* values, the real stoichiometry of the Fe1.5WO_*x*_, Fe1.0WO_*x*_ and Fe0.1WO_*x*_ materials was determined using the Fe/W atomic ratios obtained by ICP-AES. The O content, determined by TGA analysis, was calculated considering the difference in the final mass between the TGA carried out in air, where the material ends up completely oxidized (FeWO_4_), and the TGA carried out in N_2_ where the material maintains the oxygen vacancies.^46^

The position of the conduction band can also be determined through Cyclic Voltammetry (CV) measurements. When subjected to a negative potential sweep, the materials exhibit current responses corresponding to electron injection into the conduction band.^47,48^ Cyclic voltammetry measurements were performed by using a three-electrode system in a 0.2 M solution of tetrabutylammonium hexafluorophosphate (TBAPF_6_) electrolyte. An Ag/AgCl (1 M) electrode was used as the reference electrode, a gold wire served as the counter electrode, and a platinum electrode was employed as the working electrode. A volume of 20 μL of a FeWO_*x*_ dispersion in cyclohexane (20 mg/mL) was drop-cast on the platinum surface and allowed to dry at ambient conditions, resulting in the immobilization of the material. Voltammograms were recorded at 50 mV/s between −1.1 V and +1.8 V vs Ag/AgCl using a Gamry Interface 1010E potentiostat. Potentials were referenced to the Normal Hydrogen Electrode (NHE) scale by introducing ferrocene (Fc) as an internal standard, whose Fe^2+^/Fe^3+^ redox couple is set at +0.64 V on the NHE scale.^49^

### 2.5 Photocatalytic Rifampicin Degradation

The photocatalytic activities of the as-prepared FeWO_*x*_ nanoparticles were evaluated by the degradation of rifampicin under solar irradiation using a 10500 ABET solar simulator. This equipment has a xenon arc lamp with an AM 1.5G filter that provides a broadband spectrum similar to the sun, emitting radiation ranging from UV to IR. The distance from the light source to the reactor was adjusted in such a way that the intensity of the light received at the base of the reactor was 1040 W/m^2^ (1 sun). During the photodegradation studies, the temperature was kept stable at 25 °C by the introduction of the reactor in a cooling bath water. The solutions of rifampicin are colored (orange), and their concentration can be easily followed through their absorption peaks at 335 and 476 nm. To study the evolution of rifampicin degradation, the rifampicin concentration was monitored. To do that, 0.75 mL aliquots were taken every 20 min. Each aliquot was centrifuged (5 min at 15,000 rpm) to remove any traces of the photocatalyst, and the absorbance of the supernatant was analyzed using a JASCO V-670 UV–vis spectrophotometer at 335 nm. The concentration of the rifampicin antibiotic is related to the absorbance by the Lambert–Beer Law (Figure S1).

In the photodegradation studies, 25 mL of a 10 ppm aqueous solution of rifampicin (pH 7.5) was mixed with 10 mg of the photocatalyst (0.4 mg of photocatalyst per mL of rifampicin solution). The solution was kept in the dark under continuous magnetic stirring for establishing sorption equilibrium. One hour later, the solution was exposed to solar simulator irradiation up to 150 min. In these conditions, the photodegradation of organic compounds can be approximated by pseudo-first-order kinetics (eq 4):

4 $$\ln\left(\frac{C_{0}}{C_{t}}\right)=k\;t$$

where *C*_0_ is the rifampicin concentration just at the moment in which the reactor is illuminated and *C*_*t*_ is the rifampicin concentration at each time during the photocatalysis test.

The total elimination percentage of rifampicin was determined with the following equation (eq 5):

5 $$\mathrm{Elimination}\;(\%)=\left(1-\frac{C_{t}}{C_{i}}\right)\;\times \;100$$

where *C_i_* is the concentration of rifampicin just before the photocatalyst is incorporated into the reactor.

To elucidate the role of the reactive species generated during photocatalysis, scavenger experiments were conducted.^50–52^ Specifically, NaCl acts as a hole (h^+^) scavenger, isopropanol is employed to assess the involvement of hydroxyl radicals (^•^OH), NaNO_3_ works as an electron (e^–^) scavenger, and ascorbic acid is used to quench superoxide radicals (^•^O_2_^–^). These experiments were performed following the same photocatalytic protocol with 2 mM solutions of each scavenger introduced concurrently with the initiation of light irradiation in the photocatalytic reactor.

## 3 Results and Discussion

The synthesis of FeWO_*x*_ nanoparticles was first attempted following the protocol described in the literature.^17,18^ That is to say, the tungsten precursor (W­(CO)_6_) was incorporated to a mixture of dodecanediol and benzyl ether at room temperature (RT). Oleylamine (OAm) and oleic acid (OA) were introduced at 120 °C, and 1 mmol of the Fe precursor (Fe­(acac)_3_) was added at 260 °C, allowing the mixture to react for 10 min at that temperature. However, following this procedure, a mixture of Fe and W oxides (WO_3_ and Fe_3_O_4_) and some amorphous material was obtained (Figure 1a).

**Figure 1:**
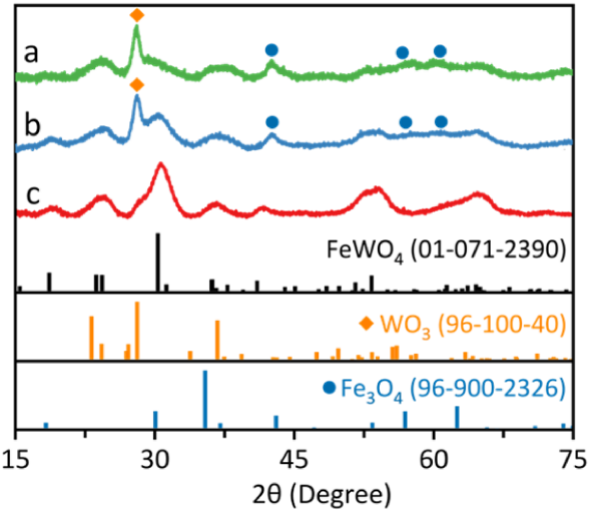
XRD patterns of the materials obtained: (a) incorporating oleic acid (OA) and oleylamine (OAm) at 120 °C and Fe­(acac)_3_ at 260 °C; (b) introducing OA and OAm at RT and Fe­(acac)_3_ added at 260 °C; and (c) incorporating OA and OAm at RT and Fe­(acac)_3_ at 170 °C.

According to the literature, tungsten oxide (WO_*x*_) can be formed from W­(CO)_6_ in a three-step process. Initially, a carbonyl group can be substituted by a coordinating ligand (for example, oleylamine) when exposed to light, leading to yellow-colored compounds (Step 1, Scheme 1).^53,54^ This first substitution favors subsequent CO replacement by alcohol-alkoxy ligands (Step 2, Scheme 1). The generated intermediate decomposes over 250 °C, oxidizing the metal center and leading to WO_*x*_ (Step 3, Scheme 1).^53,55–58^

**Scheme 1:**
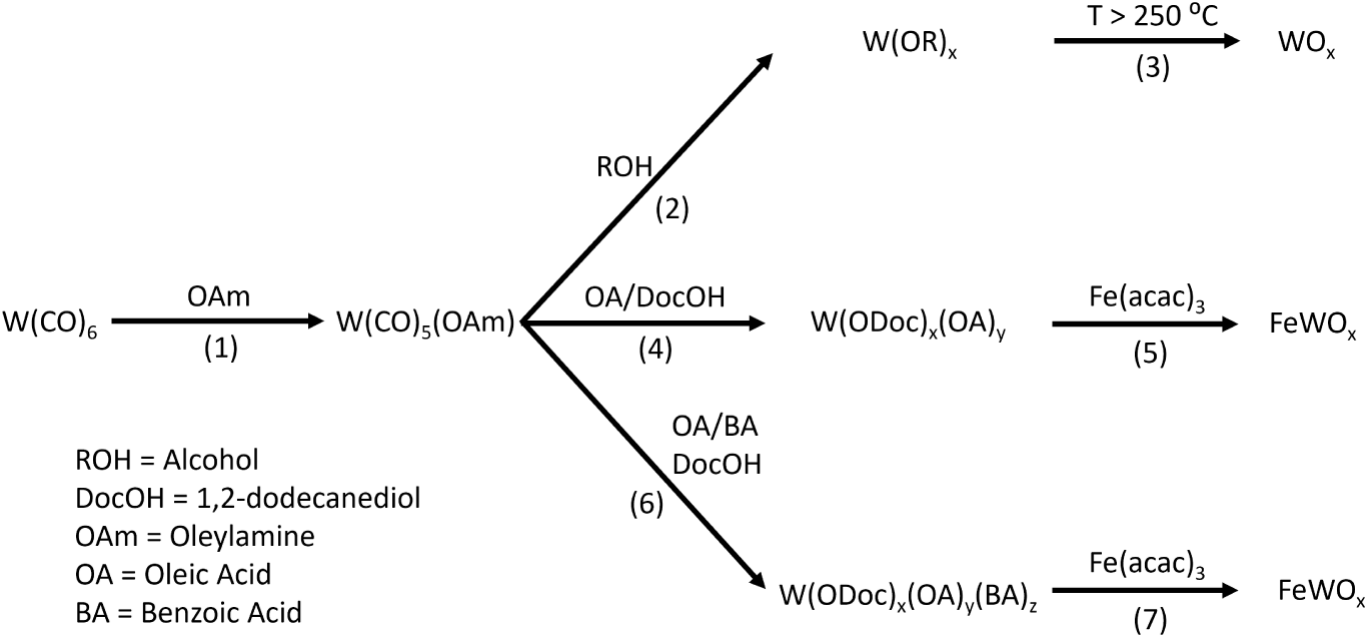
Proposed Synthetic Routes to Produce FeWO_*x*_ and WO_*x*_ from W­(CO)_6_

From this reaction route, it was hypothesized that the proposed reaction to form FeWO_*x*_ follows a similar path in three main steps. In the first step, oleylamine substitutes one CO ligand of W­(CO)_6_ (Step 1, Scheme 1). Indeed, a yellow color is observed immediately after the addition of OAm (Figure S2). During the second step, alkoxy groups from 1,2-dodecanediol and carboxyl groups from oleate ligands stabilize the W centers (Step 4, Scheme 1). It is likely that the combination of alkoxy and oleate ligands leads to a more stable intermediate that decomposes over 250 °C. In the third step, these decomposing tungsten intermediates are able to react with the iron precursor, leading to FeWO_*x*_ instead of forming WO_*x*_ (Step 5, Scheme 1).

Keeping this mechanism in mind, the synthetic method was adjusted. Initially, the modifications focused on the OA/OAm pair. These species are classified as hard Lewis bases and have the ability to coordinate with metal centers; therefore, they can be used to stabilize the metal precursors and prevent nanoparticle aggregation.^59–61^

It was considered that adding the OA and OAm ligands at RT, at the beginning of the reaction, instead of adding them at 120 °C, could be a good way to stabilize the tungsten reactive species^46,62,63^ and facilitate the beginning of the reactions. Indeed, when adding OA and OAm at RT, a crystalline FeWO_*x*_ phase could be obtained but still with the presence of WO_3_ and Fe_3_O_4_ (Figure 1b).

According to previous studies,^62^ the thermal decomposition of Fe­(acac)_3_ in benzyl ether starts at 170 °C. Thus, it was decided to incorporate the Fe precursor at 170 °C (instead of 260 °C) to facilitate interaction with the tungsten intermediates at the same time they are formed. The growth of the nanomaterials was maintained at 260 °C. With the implementation of these changes, it was possible to synthesize a FeWO_*x*_ phase-pure material. Some WO_3_ traces were detected in the XRD pattern, but this is consistent with the XRD pattern reported in the literature following a similar synthetic protocol.^17,18^ Therefore, it was decided to synthesize the material incorporating the OA/OAm pair at RT and 1 mmol of Fe­(acac)_3_ at 170 °C (Figure 1c).

During the implementation of this improved synthesis, reproducibility problems were detected, as at some point an amorphous material was obtained for every synthesis (Figure S3). To achieve a crystalline material again, the amount of OA was increased from 3 to 9 mmol, aiming at enhancing the stability of the W intermediates. In that case, there were enough ligands to complete the coordination sphere of Fe and W, and the total positive charges of the W^6+^ and Fe^3+^ pair could be neutralized with the negative charges of the oleate ligands. XRD results (Figure 2a) show the successful formation of FeWO_*x*_ although there were still impurities of WO_3_ and Fe_3_O_4_ in the material. Increasing the amount of OA to 18 mmol led to a similar result (Figure 2a). According to the ICP-AES analysis (Table S1), as the amount of OA increases, more W was incorporated into the final material. Consequently, increasing the amount of coordinating ligands (OA) is a good strategy to improve the stabilization of the reactive tungsten species. However, only increasing the amount of OA is not enough to stabilize a suitable W intermediate that reacts with the Fe precursor to produce FeWO_*x*_.

**Figure 2:**
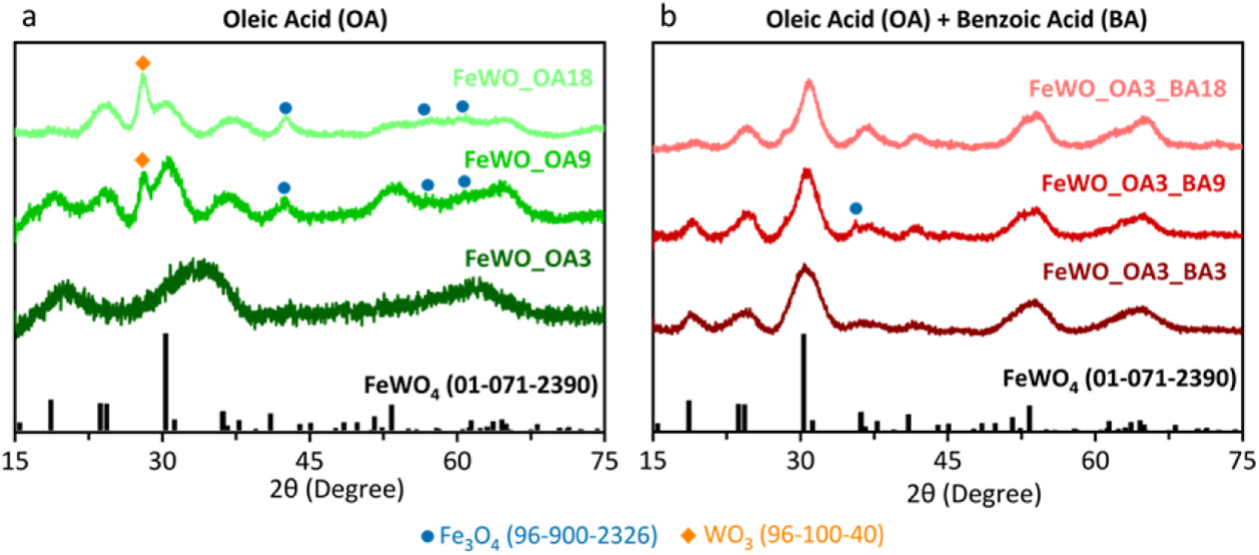
XRD patterns of the materials obtained using (a) different amounts of OA or (b) a mixture of 3 mmol of OA and different quantities of BA.

Apparently, it is necessary to introduce an additional ligand that is able to stabilize the reactive tungsten species until the reaction with iron and before decomposing to form WO_*x*_. According to some literature reports, the use of benzyl ether (BE) as a solvent can cause problems in the syntheses, such as an increase in the polydispersity of nanoparticle sizes, low reproducibility, and poor control of the product’s stoichiometry.^19–22^ Specifically, BE is prone to oxidation and can lead to the formation of benzaldehyde (BZ), benzyl benzoate (BB), and/or benzoic acid (BA).^19,23,64^ These molecules (BZ, BB, or BA) could play a role in the reaction; therefore, it was decided to intentionally add them to the reaction medium. When 18 mmol of benzaldehyde (BZ) or benzyl benzoate (BB) were added, a mixture of FeWO_*x*_ with a significant amount of Fe_3_O_4_ was obtained (Figure S4). On the other hand, adding 18 mmol of benzoic acid (BA) delivered crystalline FeWO_*x*_ (Figure 2b). Also, if 3 and 9 mmol of BA were added, mainly the FeWO_*x*_ compound is obtained, but in lower quantities (85 mg of FeWO_OA3_BA3, 105 mg of FeWO_OA3_BA9, and 140 mg of FeWO_OA3_BA18, Table S1). In addition, traces of magnetite are detected in the XRD pattern (Figure 2b) when 9 mmol of BA were incorporated. Therefore, the addition of 18 mmol of benzoic acid is an adequate amount to direct the reaction preferentially toward the formation of FeWO_*x*_. Moreover, according to ICP-AES analyses (Table S1), when 18 mmol of BA are added, the amount of W incorporated into the material is practically double than when adding 3 or 9 mmol, suggesting that BA coordinates with W metal centers, stabilizing the reactive tungsten species for their reaction with Fe to form FeWO_*x*_ (Steps 6 and 7, Scheme 1).

To further confirm the role of benzoic acid as a necessary molecule to obtain FeWO_*x*_ nanoparticles, a reaction replacing benzyl ether with 1-octadecene (ODE) was carried out. If benzoic acid was not added, then Fe_3_O_4_ was obtained as the predominant phase (Figure S5a). However, when 18 mmol of benzoic acid were added, the FeWO_*x*_ phase was obtained (Figure S5b), although in very low yield. These results further confirmed that 1-octadecene can replace benzyl ether as the reaction solvent and that the presence of benzoic acid as a coordinating ligand is crucial for the proper stabilization of reactive W species and the subsequent formation of FeWO_*x*_.

Some authors have found reproducibility problems when using benzyl ether as a solvent^19–22^ and have suggested that these can be caused by oxidation products (mostly benzaldehyde and benzyl benzoate) formed during synthesis at high temperatures. In our case, when using a batch that had formed benzoic acid during storage, the W intermediates could be stabilized, and crystalline FeWO_*x*_ could be obtained (Figures 1b and S3a). However, when using a fresh batch, benzoic acid must be incorporated on purpose to obtain the desired material. According to our results, the issues caused by benzyl ether can be related to the presence or absence of byproducts (not only benzaldehyde or benzyl benzoate but also benzoic acid) formed upon storage (not only during the synthesis). We believe this result may be of interest to other researchers using benzyl ether-based synthesis.

To check whether the presence of OA is still necessary to obtain FeWO_*x*_, the reaction was carried out without adding oleic acid and modifying the amount of benzoic acid (3, 9, and 18 mmol). When only 3 mmol of benzoic acid were added, an amorphous material with traces of Fe_3_O_4_ was detected in the XRD pattern (Figure S6). In the FeWO_BA9 material, mainly the crystalline phase FeWO_*x*_ was obtained with traces of Fe_3_O_4_, like in the FeWO_OA3_BA9 synthesis (Figure 2b). Finally, only the FeWO_*x*_ crystalline phase was obtained in the FeWO_BA18 synthesis, like in the FeWO_OA3_BA18 one, but the quantity was lower (90 mg vs 140 mg, Table S1). These results confirm that a minimum number of ligands is needed to generate stable W intermediates, and the synergistic effect of OA and BA to increasing the reaction yield. Therefore, for the subsequent studies, 18 mmol of BA and 3 mmol of OA were added to the synthesis medium (FeWO_OA3_BA18).

The Raman spectrum of FeWO_OA3_BA18 (Figure 3a) shows a signal at 125 cm^–1^ induced by the lattice vibration.^11,12^ The bands at 217 cm^–1^ and 278 cm^–1^ can be assigned to the bending vibration of the Fe–O–W modes.^11,65^ The signal at 364 cm^–1^ is caused by the ν_2_ bending mode,^11^ the band at 660 cm^–1^ results from an antisymmetric bridging mode relating to the tungstate chain,^11^ and the strongest signal at 877 cm^–1^ originates from the ν_1_ symmetric A_g_ mode of terminal O–W–O.^11,12,28,65^

**Figure 3:**
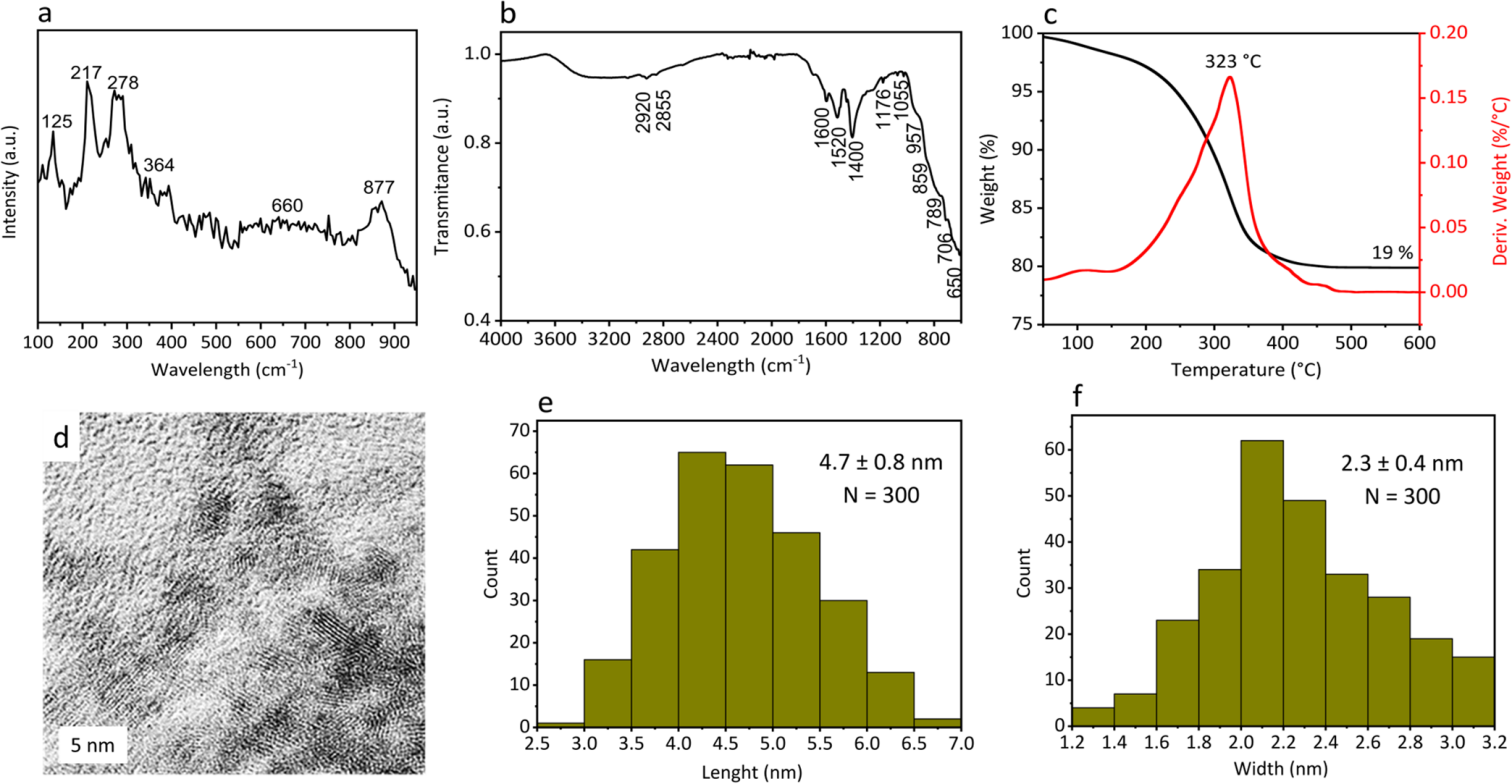
Characterization of FeWO_OA3_BA18: (a) Raman spectroscopy, (b) FTIR spectroscopy, (c) TGA analysis, and (d) TEM image and histograms of (e) length and (f) width.

Figure 3b shows the FTIR spectrum of the material recorded between 600 cm^–1^ and 4000 cm^–1^. The signals at 600–720 cm^–1^ are due to the stretching and bending modes of the W–O bonds.^12,16,66^ The band at 789 cm^–1^ may be due to the W–O vibration mode.^12,16,66^ The signal at 859 cm^–1^ is attributed to symmetric vibrations of bridging oxygen atoms of Fe–O–W.^12,16^ The intense bands at 1400 cm^–1^ and 1520 cm^–1^ could be BA and OA carbonyl groups bonded to the metal centers. The bands at 1600 cm^–1^ and 3600–3000 cm^–1^ are bending and stretching of O–H bonds, respectively.^4,67^ Additionally, the signals around 2800–3000 cm^–1^ are typical of C–H bond stretching vibrations. These last bands can be attributed to the ligands that stabilize the nanoparticles, contributing to 19% of the material’s weight, according to the thermogravimetric analysis (TGA). The calcination of this organic part occurs mainly at 323 °C (Figure 3c).

The TEM images (Figure 3d) show slightly elongated nanosheets with a length of 4.7 ± 0.8 nm (Figure 3e) and a width of 2.3 ± 0.4 nm (Figure 3f), in agreement with the crystallite size obtained from XRD using the Scherrer equation (4 nm). Some crystalline planes can also be detected (Figure 3d), and it is observed that the nanoparticles tend to agglomerate. Electron Energy Dispersive X-ray spectroscopy (EDX) performed during SEM and TEM (Figure S7a) analysis shows an excess of Fe over W (Fe/W ratio = 1.5 ± 0.3 from EDX-SEM and 1.4 ± 0.2 from EDX-TEM) in agreement with the Fe excess detected in the ICP-AES analysis (Fe/W = 1.4 ± 0.01, Table S1).

The N_2_ adsorption–desorption Brunauer–Emmett–Teller (BET) isotherm (Figure S8a) corresponds to a type IV isotherm with an H3 hysteresis loop, characteristic of mesoporous materials formed by aggregates of lamellar nanoparticles. The BET surface area is 116 m^2^/g, with a pore volume of 0.09 cm^3^/g and an average pore size of 3.1 nm (Figure S8b). The pore size is close to the nanoparticle size determined by the Scherrer equation and observed in the TEM pictures, suggesting that the porosity could be related to the interparticle space. Interestingly, this compound shows a larger BET surface area, smaller pore volume, and similar pore size than other FeWO_4_ nanomaterials reported in the literature (BET surface area: 35 or 63 m^2^/g; pore volume: 0.223 or 0.31 cm^3^/g; and pore size: 2.2 or 3.7 nm).^2,68^ Additionally, the specific surface area is also higher than WO_3_ nanosheet-based materials (13–55 m^2^/g).^69–71^

X-ray Photoelectron Spectroscopy (XPS) analyses (Figure 4) show the presence of C (49.85% at.), O (35.12% at.), Fe (8.72% at.), and W (7.31% at.), again pointing to some excess of iron over tungsten (Fe/W = 1.2). With these results, it can be confirmed that the compound is not stoichiometric. The C 1s spectrum (Figure 4b) shows three components (284.80, 285.97, and 288.72 eV), assigned respectively to C–C/C–H, C–O, and C=O, and corresponding to the organic ligands stabilizing the nanoparticles. The O 1s narrow spectra (Figure 4c) can be fitted to three components at 530.55 eV, 531.57 eV, and 532.66 eV attributed to W–O–W, Fe–O–Fe, and Fe–O, respectively.^11,72,73^

**Figure 4:**
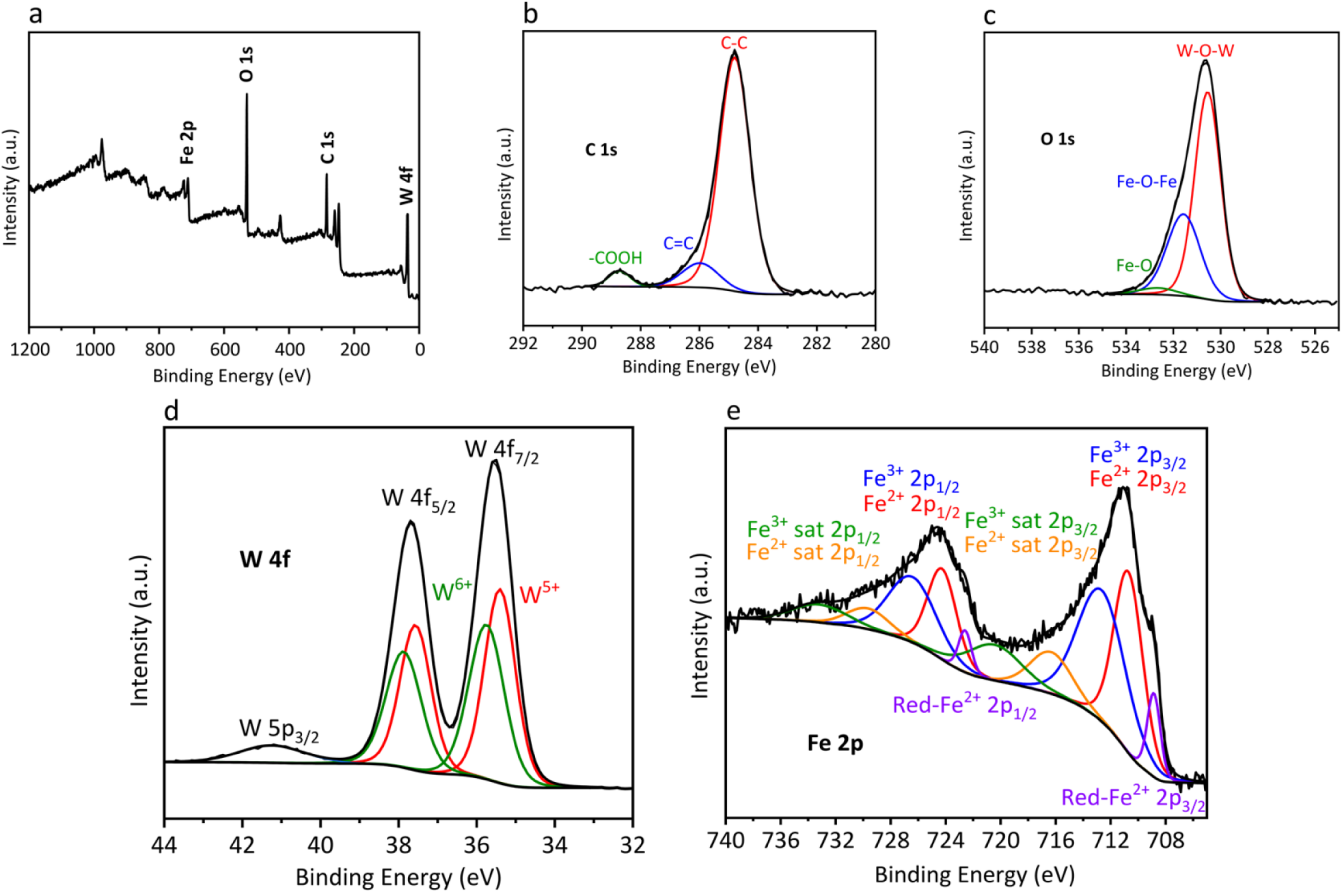
(a) General XPS spectrum of FeWO_OA3_BA18 and their high-resolution XPS spectra: (b) C 1s, (c) O 1s, (d) W 4f, and (e) Fe 2p.

The high-resolution XPS spectrum of W 4f (Figure 4d) consists of two peaks: W 4f_7/2_ and W 4f_5/2_. Also, the W 5p_3/2_ peak can be observed at 41.5 eV, which is assigned an RSF of 0 to perform quantifications. Each of the W 4f_7/2_ and W 4f_5/2_ peaks can be deconvoluted into two components: W^5+^ (35.40 and 37.57 eV) and W^6+^ (35.75 and 37.87 eV).^17,18,74–78^

The high-resolution XPS spectrum of Fe 2p (Figure 4e) shows a component that can be attributed to Fe^2+^ species (710.71 and 724.26 eV) with the corresponding Fe^2+^ satellites (716.33 and 729.65 eV) and another component attributed to Fe^3+^ species (712.66 and 726.42 eV) with the corresponding Fe^3+^ satellites (720.45 and 733.20 eV).^17,18,79–86^ At lower binding energies (708.86 and 722.59 eV), a third component is detected (denoted as Red-Fe^2+^). Some authors attribute this component to metallic Fe(0),^87,88^ but it appears at higher binding energies than reported Fe(0) species (∼706.6 eV).^80,89^ We believe that this component can be related to Fe^2+^ species with higher electronic density (electronic density donated by oxygen atoms) and can be related to distortions generated by iron vacancies in the lattice, or Fe^2+^ octahedral sites surrounded by W^5+^ octahedra, where W^5+^ removes less electronic density from oxygen atoms than W^6+^. The calculated W^5+^/W^6+^ and Fe^2+^/Fe^3+^ ratios are 1.10 and 0.99, respectively. In both cases, they are close to unity, with a slight excess of W^5+^ over W^6+^, as previously reported.^18^

Once the individual roles of the ligands have been elucidated and the conditions to ensure the reproducibility of the synthesis have been determined, the tailored synthesis of FeWO_*x*_ was explored. In this study, different FeWO_*x*_ materials have been synthesized with several nominal Fe/W atomic ratios (0.1, 0.3, 0.5, 1.0, and 1.5), with the aim of determining how these ratios affect the physical–chemical properties of these compounds. From here, the notation of the compounds will be Fe*a*WO_*x*_, where *a* is the mmol of Fe (0.1, 0.3, 0.5, 1.0, and 1.5 mmol) per mmol of W added to the reaction.

In all cases, the formation of the FeWO_*x*_ phase was observed in the XRD patterns (Figure 5a). Also, a small shoulder at 27° can be detected for all compounds, except Fe1.5WO_*x*_, which can be attributed to a little amount of WO_3_ formed during the synthesis under stoichiometric or Fe defect conditions. The crystallite size obtained using the Scherrer equation is close to 4 nm for all materials.

**Figure 5:**
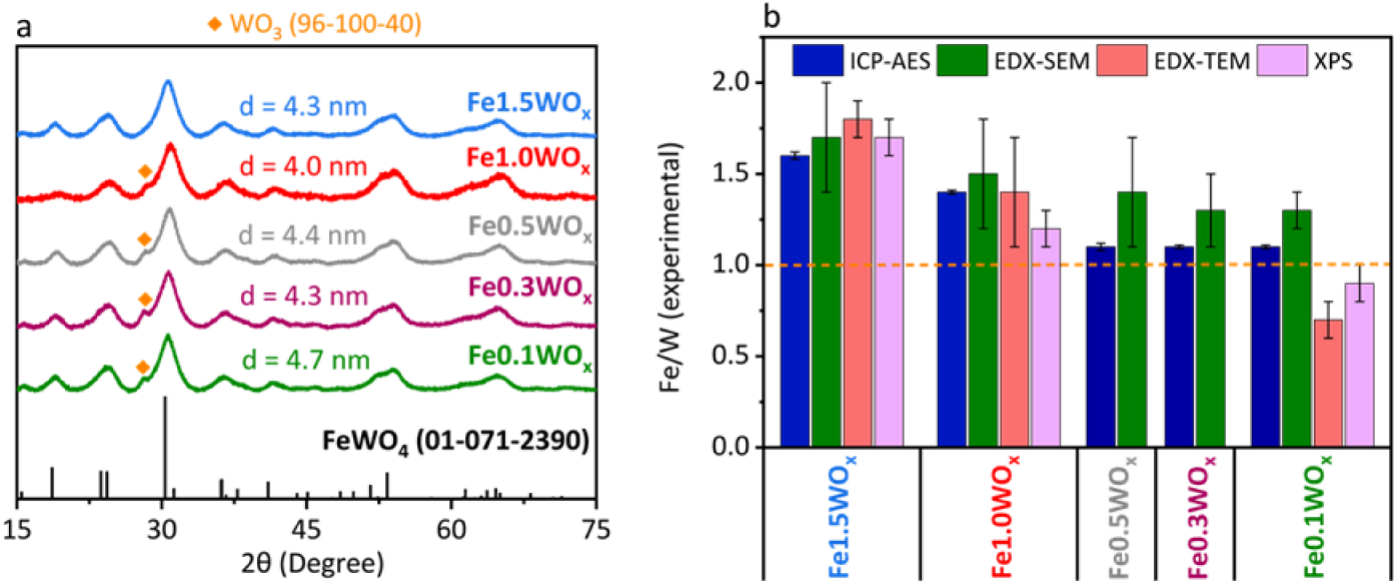
(a) XRD patterns and crystal size dimensions obtained by applying the Scherrer equation to the 31° peak of the materials with different nominal ratios. (b) Representation of the nominal Fe/W atomic ratio versus the Fe/W ratio from ICP-AES (blue), EDX-SEM (green), EDX-TEM (red), and XPS (purple).

ICP-AES, EDX-SEM, EDX-TEM, and XPS results confirm that the Fe/W ratio in the material can be tuned by modifying the nominal Fe/W ratio (Figure 5b and Table S2). In this way, it is possible to obtain three families of materials with (i) Fe excess (Fe/W = 1.6 ± 0.02 for Fe1.5WO_*x*_), materials with a (ii) slight Fe excess (Fe/W = 1.4 ± 0.01 for Fe1.0WO_*x*_), and a third one (iii) with a Fe/W ratio close to 1 (Fe/W = 1.1 ± 0.02, 1.1 ± 0.01, and 1.1 ± 0.01 for Fe0.5WO_*x*_, Fe0.3WO*_x_,* and Fe0.1WO_*x*_). In view of these results, Fe1.5WO_*x*_, Fe1.0WO*_x_,* and Fe0.1WO_*x*_ were selected as representative examples of each family of materials.

TEM images of Fe0.1WO_*x*_ (Figure S7d), and Fe1.5WO_*x*_ (Figure S7g) were obtained revealing the presence of slightly elongated nanoparticles with well-defined crystalline planes and exhibiting dimensions comparable to those observed in Fe1.0WO_*x*_ (i.e., FeWO_OA3_BA18; Figure 3d). Specifically, Fe0.1WO_*x*_ shows an average length of 4.5 ± 0.9 nm and a width of 2.1 ± 0.5 nm, while Fe1.5WO_*x*_ exhibits dimensions of 4.4 ± 0.9 nm in length and 2.1 ± 0.4 nm in width, consistent with the crystallite sizes estimated by using the Scherrer equation (Figure 5a).

Energy-Dispersive X-ray Spectroscopy performed during TEM (EDX-TEM) revealed a trend in the Fe/W atomic ratio consistent with that observed by using other techniques, with values of 1.8 ± 0.1 for Fe1.5WO_*x*_, 1.4 ± 0.2 for Fe1.0WO_*x*_, and 0.7 ± 0.1 for Fe0.1WO_*x*_ (Figure 5b). A similar trend was observed in EDX-SEM analyses, which showed Fe/W atomic ratios of 1.7 ± 0.3 for Fe1.5WO_*x*_, 1.5 ± 0.3 for Fe1.0WO_*x*_, and 1.3 ± 0.1 for Fe0.1WO_*x*_.

The N_2_ adsorption–desorption experiments (Figure S8 and Table 2) show that the materials with lower Fe/W atomic ratios have a greater BET area surface and higher pore volumes. A lower iron content can result in iron vacancies, leading to form more open structures and increased porosity.^90^ Again, the pore diameter, close to 4 nm, is similar to the particle size determined by the Scherrer equation and observed in TEM pictures, pointing to a porosity related to interparticle space.^91,92^

**Table 2:** BET Surface Area, Pore Volume, and Pore Diameter of Compounds Fe0.1WO_*x*_, Fe1.0WO*_x_,* and Fe1.5WO_*x*_

Compound	*S*_BET_ (m^2^/g)	Pore volume (cm^3^/g)	Pore diameter (nm)
Fe0.1WO_x_	108	0.115	4.2
Fe1.0WO_x_	116	0.090	3.1
Fe1.5WO_x_	62	0.071	4.6

The Raman and FTIR spectra (Figure S9) are very similar to the Fe1.0WO_*x*_ material (Figure 3a,b), with some minor differences. In the Raman spectrum, the peaks at 217 cm^–1^ and 278 cm^–1^, assigned to the bending vibration of the Fe–O–W modes, are less intense for Fe0.1WO_*x*_, while the peaks 660 cm^–1^ and 877 cm^–1^, related to antisymmetric bridging mode of tungstate chains and terminal O–W–O bonds, respectively, are more intense in this material.^11,12,65^ Similarly, in the FTIR spectrum of Fe0.1WO_*x*_, the bands observed in the 600–720 cm^–1^ region, corresponding to the stretching and bending modes of W–O bonds, along with the band at 789 cm^–1^, attributed to W–O vibrational modes, exhibit increased intensity.^12,16,66^

XPS analyses show that both the Fe1.5WO_*x*_ and Fe0.1WO_*x*_ samples present Fe/W atomic ratios (1.7 ± 0.1 and 0.9 ± 0.1, respectively) similar to those obtained by SEM-EDX, TEM-EDX, and ICP-AES (Table S2). The XPS peaks of these compounds (Figure S10) are located at similar positions to those of the Fe1.0WO_*x*_ sample (Figure 4). In all cases, the W^5+^/W^6+^ ratio is close to unity (1.0–1.1). Similarly, the Fe^2+^/Fe^3+^ ratio is approximately unity for the Fe1.0WO_*x*_ and Fe1.5WO_*x*_ compounds (1.0 and 1.2, respectively), whereas for Fe0.1WO*_x_,* the ratio is 1.9. Additionally, the relative amount of the *Red-Fe* ^*2+*^ component is higher in this material (10.6%) than in Fe1.0WO_*x*_ and Fe1.5WO_*x*_ (6.7% and 3.2%, respectively). The higher amount of iron-reduced species (Fe^2+^ and *Red-Fe* ^*2+*^) in the Fe0.1WO_*x*_ material can be a result of the relatively lower ratio of the Fe­(III) precursor (Fe­(acac)_3_) per reducing species in the reaction medium (oleylamine and W(0) in W­(CO)_6_).

The optical band gaps were obtained through their Tauc plots (Figure S11).^93^ The calculated band gaps were 2.06 eV, 1.90 eV, and 1.45 eV for Fe1.5WO_*x*_, Fe1.0WO*_x_,* and Fe0.1WO_*x*_, respectively, in agreement with those reported in the literature for FeWO_4_ materials (1.8–3.0).^11,13–16^ The lower iron content material (Fe0.1WO_*x*_) shows a remarkably reduced band gap (0.6 eV less) with potential for absorbing more photons of the solar spectrum. In short, modifying the Fe/W atomic ratio of these compounds alters their optical band gap, which can be very attractive in photocatalytic applications.

The energy positions of conduction (CB) and valence (VB) bands of a semiconductor material can be estimated using empirical equations (eqs 2 and 3 in Section 2),^43–45^ knowing their band gap and their real stoichiometry that can be extracted from ICP analysis and thermogravimetric analyses (TGAs) performed in oxygen and nitrogen (Figure S12). The real stoichiometry of Fe0.1WO_*x*_, Fe1.0WO*_x_,* and Fe1.5WO_*x*_ was Fe_1.1_WO_3.6_, Fe_1.4_WO_3.9_, and Fe_1.6_WO_3.4_, respectively. Consequently, the energy positions of the VB were 2.53, 2.71, and 2.61 V vs NHE for Fe0.1WO_*x*_, Fe1.0WO_*x*_, and Fe1.5WO_*x*_, respectively. Correspondingly, the CB positions were found to be 1.08, 0.81, and 0.55 V vs NHE for Fe0.1WO_*x*_, Fe1.0WO_*x*_, and Fe1.5WO_*x*_, respectively (Figure 6).

**Figure 6:**
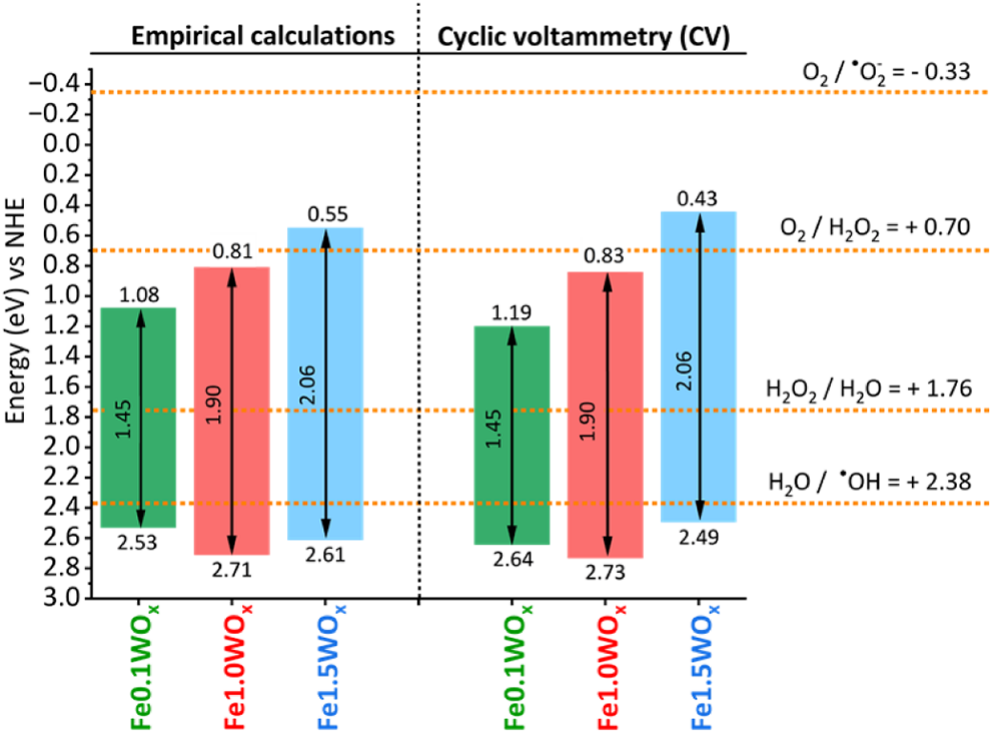
Energy positions of the valence and conduction bands of compounds Fe0.1WO_*x*_, Fe1.0WO*_x_,* and Fe1.5WO_*x*_, obtained through (a) empirical calculations and (b) cyclic voltammetry.

In addition, the estimated conduction band (CB) determined through Cyclic Voltammetry (CV) measurements (Figure S13) was 1.19, 0.83, and 0.43 V vs NHE for Fe0.1WO_*x*_, Fe1.0WO_*x*_, and Fe1.5WO_*x*_, respectively. Subsequently, using the optical band gap (*E*_g_) of each material, the valence band energy positions were calculated to be 2.64, 2.73, and 2.49 V for Fe0.1WO_*x*_, Fe1.0WO_*x*_, and Fe1.5WO_*x*_, in that order. The energy band positions derived from CV (Figure 6) agree well with the energy levels derived from empirical calculations and are consistent with those previously reported in the literature.^15,16^

The photocatalytic activity of Fe0.1WO_*x*_, Fe1.0WO*_x_,* and Fe1.5WO_*x*_ was examined by evaluating the degradation of rifampicin antibiotic solutions (pH 7.5) as a model of emerging pollutants in aqueous media. The zeta potentials of the materials at pH 7.5 are −27.9 ± 0.8 mV, −24.9 ± 0.4 mV, and −14.1 ± 0.7 mV (Fe0.1WO_*x*_, Fe1.0WO*_x_,* and Fe1.5WO_*x*_ respectively), and under these conditions, the rifampicin molecule is electrically neutral (p*K*_1_ = 6.9 and p*K*_2_ = 7.5); therefore, electrical repulsion between the pollutant molecule and the materials is not expected.

Just after the dark stage, when the adsorption–desorption equilibrium between the photocatalyst and the contaminant was established, the reactor was subjected to irradiation by a solar simulator. In 150 min, Fe0.1WO_*x*_, Fe1.0WO*_x_,* and Fe1.5WO_*x*_ photodegraded 67%, 25%, and 27% of rifampicin, respectively (Figure 7a).

**Figure 7:**
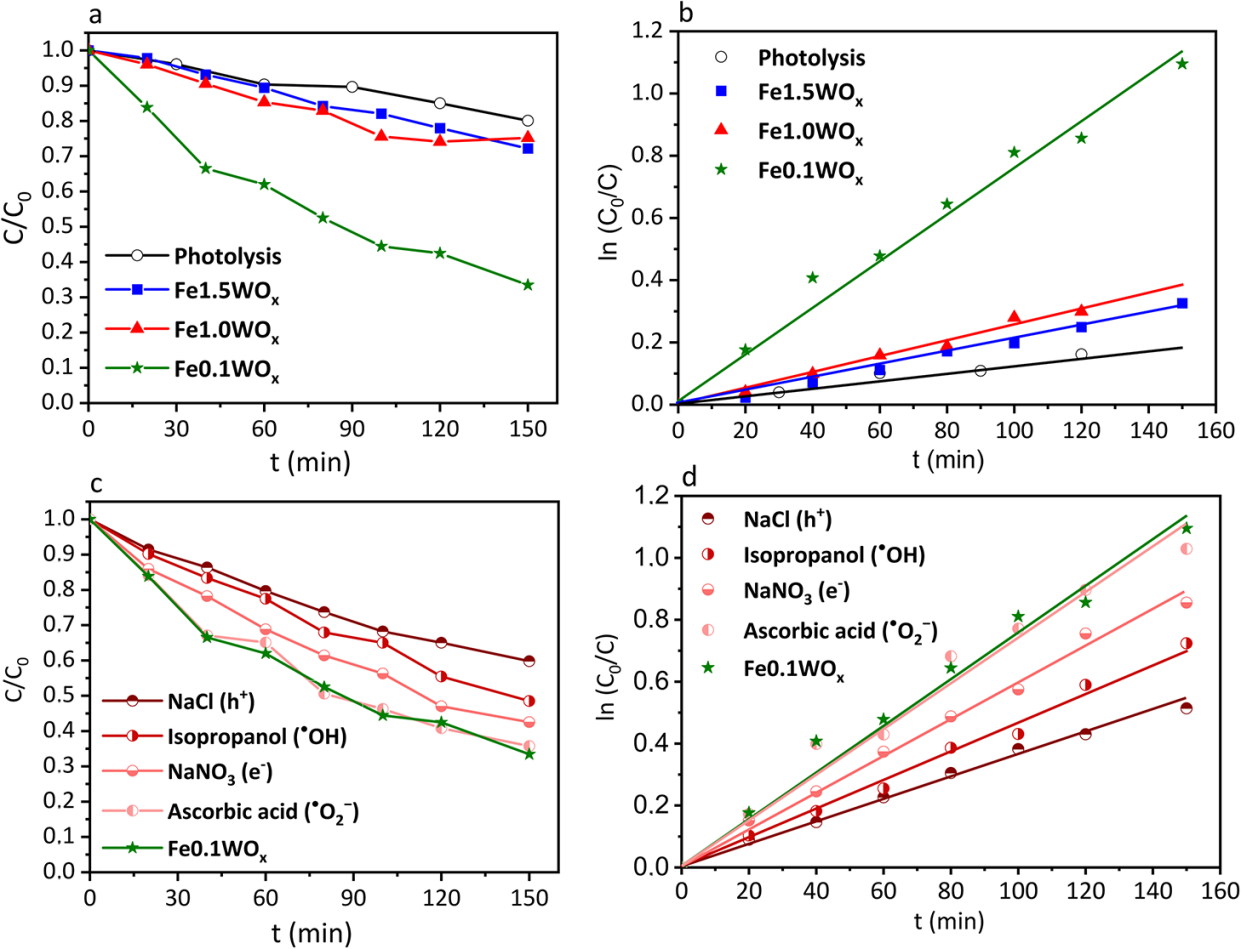
(a) Rifampicin photodegradation experiments with Fe1.5WO_*x*_ (blue), Fe1.0WO_*x*_ (red), and Fe0.1WO_*x*_ (green) photocatalysts. Each experiment was performed with 0.4 mg of the photocatalyst per mL of solution at 25 °C and pH 7.5. *C*_0_ = 10 ppm. (b) Pseudo-first-order kinetic representations.

All reaction mechanisms can be accurately described by first-order kinetics (Figure 7b), with Fe0.1WO_*x*_ showing a 3-fold fastest constant than the other compounds (0.0076 min^–1^, 0.0023 min^–1^, and 0.0021 min^–1^ for Fe0.1WO_*x*_, Fe1.0WO*_x_,* and Fe1.5WO_*x*_ respectively, Table S3). These differences can be attributed to the smaller band gap of Fe0.1WO_*x*_ (1.45 eV) as compared to Fe1.5WO_*x*_ and Fe1.0WO_*x*_ (2.06 and 1.90 eV), making it able to absorb more photons.

As compared to similar systems reported in the literature (Table 3), Fe0.1WO_*x*_ does not reach the high degradation rates (90–97%) reported for some Fe_2_O_3_ and FeWO_4_ nanoparticles. However, those studies focus on molecules that are simpler than rifampicin or use UV irradiation. On the other hand, Fe0.1WO_*x*_ exhibits a higher photocatalytic performance than other WO_3_, Fe_2_O_3_, and FeWO_4_ nanomaterials, especially those focusing on the degradation of antibiotics (ciprofloxacin, levofloxacin, or rifampicin).

**Table 3:** Photocatalytic Activity of WO_3_, Fe_2_O_3_, and FeWO_4_ Nanomaterials Compared with Those Reported in This Study^a^

Material	Pollutant	Light source	*C*_0_ (ppm)	*m*/*V* (g/L)	*t* (min)	Degradation (%)	ref.
WO_3_	Rhodamine B	300 W Xe lamp	10	0.25	120	22	^95^
WO_3_	Ciprofloxacin	300 W Xe lamp	10	4	120	8	^71^
WO_3_	Levofloxacin	300 W Xe lamp	20	1	120	50	^69^
Fe_2_O_3_	Rifampicin	500 W halogen lamp	20	0.2	80	22	^96^
Fe_2_O_3_	Methyl orange	250 W halogen lamp	100	0.2	100	94	^94^
FeWO_4_	Rhodamine B	4 W LED	5	0.8	120	3	^29^
FeWO_4_	Rhodamine B	300 W Xe lamp	5	0.3	55	90	^30^
FeWO_4_	Methyl orange	UV–visible light irradiation	10	1	120	97	^11^
FeWO_4_	Malachite green	UV irradiation Pen-Ray Lamps Group Type 1115 (25 W, 18 mA, 254 nm)	20	0.25	120	90	^4^
*Fe0.1WO*_*x*_	Rifampicin	Solar simulator	10	0.4	120	65	This work
*Fe1.0WO*_*x*_	Rifampicin	Solar simulator	10	0.4	120	25	This work
*Fe1.5WO*_*x*_	Rifampicin	Solar simulator	10	0.4	120	27	This work

a Initial pollutant concentration (*C*_0_), concentration of the catalyst (*m*/*V*), reaction time (*t*), and % degradation.

To investigate the role of h^+^, e^–^, ^•^O_2_^–^, and ^•^OH reactive species in the degradation of rifampicin, specific scavengers (NaCl, NaNO_3_, ascorbic acid, and isopropanol, respectively) were employed. These agents can selectively react with targeted reactive species slowing down the photocatalytic process. The Fe0.1WO_*x*_ material was selected for these studies, as it exhibited the highest photocatalytic performance among the tested catalysts.

The addition of 2 mM ascorbic acid, NaNO_3_, isopropanol, and NaCl reduced rifampicin degradation from 76% to 64%, 57%, 51%, and 40%, respectively (Figure 7c). Correspondingly, the kinetic rate constants (Figure 7d) in the presence of these scavengers were 0.0074 min^–1^, 0.0060 min^–1^, 0.0047 min^–1^, and 0.0023 min^–1^, for ascorbic acid, NaNO_3_, isopropanol, and NaCl, respectively (Table S4). These results indicate that the role of electrons in the conduction band is minimal in the photodegradation processes. The addition of ascorbic acid (^•^O_2_^–^ scavenger) does not alter the reaction, and the addition of NaNO_3_ (e^–^ scavenger) minimally reduces the kinetic constant. From Figure 6 it is clear that the position of Fe0.1WO_*x*_ conduction is not suited for superoxide radical generation, and apparently, though possible, the direct transfer of electrons from the conduction band to the rifampicin molecule might not be the main photodegradation path. On the contrary, the addition of isopropanol (^•^OH radical scavenger) and especially NaCl (h^+^ scavenger) dramatically reduces the degradation ability of Fe0.1WO_*x*_, thus pointing to the direct transfer of holes from Fe0.1WO_*x*_ to the rifampicin molecule as the main photodegradation path, followed by the generation of ^•^OH radicals.

## 4 Conclusions

The synthesis of nonstoichiometric FeWO_*x*_ nanoparticles via thermal decomposition in benzyl ether has been successfully optimized and standardized. The individual roles of the chemicals involved in the synthesis have been elucidated, enabling precise control over the reaction conditions and markedly improving the reproducibility of the process. Importantly, we have identified benzoic acid, a benzyl ether oxidation byproduct formed during solvent storage, as a necessary ligand for stabilizing tungsten intermediates and enabling consistent FeWO_*x*_ formation. This insight could prove valuable for other researchers working with benzyl ether as a solvent.

Furthermore, this synthetic strategy enables the tuning of the iron content in the final FeWO_*x*_ materials by adjusting the Fe/W atomic ratio in the reaction mixture. As a result, a series of FeWO_*x*_ compounds with a very high or slightly defective iron content were obtained, showing distinct physicochemical properties. Lowering the Fe/W ratio led to a material with a Fe/W ratio close to unity that showed an increased specific surface area (up to 116 m^2^/g), a narrower band gap (≈1.4 eV), and superior photocatalytic performance in the degradation of rifampicin.

Scavenger experiments confirmed that the main degradation paths exploit the photogenerated holes in the valence band by directly transferring the holes to the pollutant molecule or by generating hydroxyl radicals (^•^OH).

## Supplementary Material



## Notes

Calibration curve for rifampicin antibiotic according to absorbance values at 335 nm (Figure S1); color changes observed in the reaction crude of FeWO*_x_* after the addition of oleylamine (Figure S2); representative XRD patterns of the materials obtained before and after the reproducibility problems of the synthesis (Figure S3); Fe/W ratios, concentration of Fe and W, mg of material obtained, and % of organic content (Table S1); XRD pattern of the materials obtained adding benzyl benzoate or benzaldehyde (Figure S4); XRD patterns obtained using 1-octadecene as the solvent with and without the addition of benzoic acid (Figure S5); XRD patterns of the materials obtained without incorporating oleic acid and using different quantities of benzoic acid (Figure S6); EDX-TEM spectra of Fe1.0WO_*x*_, Fe0.1WO*_x_,* and Fe1.5WO_*x*_, TEM images, length histograms and width histograms of Fe0.1WO_*x*_ and Fe1.5WO_*x*_ (Figure S7); BET nitrogen adsorption isotherm plots and DFT method—pore size and pore volume distribution of Fe1.0WO_*x*_, Fe0.1WO*_x_,* and Fe1.5WO_*x*_ (Figure S8); Fe/W ratios from ICP, concentration of Fe and W, Fe/W, Fe^2+^/Fe^3+^, W^5+^/W^6+^ atomic ratios from XPS, Fe/W atomic ratios from EDX-SEM, zeta potential, mg of material, and % of organic content (Table S2); Raman and FTIR spectra of Fe0.1WO_*x*_, Fe1.0WO*_x_,* and Fe1.5WO_*x*_ (Figure S9); general XPS spectra of Fe0.1WO_*x*_, Fe1.0WO*_x_,* and Fe1.5WO_*x*_ and C 1s, O 1s, Fe 2p, and W 4f detailed XPS spectra of Fe0.1WO_*x*_ and Fe1.5WO_*x*_ (Figure S10); UV–vis DRS spectra and Tauc plots of Fe0.1WO_*x*_, Fe1.0WO*_x_,* and Fe1.5WO_*x*_ (Figure S11); TGA analysis in air and N_2_ of Fe0.1WO_*x*_, Fe1.0WO*_x_,* and Fe1.5WO_*x*_ (Figure S12); cyclic voltammetries of Fe0.1WO_*x*_, Fe1.0WO*_x_,* and Fe1.5WO_*x*_ (Figure S13); pseudo-first-order kinetic constants of Fe0.1WO_*x*_, Fe1.0WO_*x*_, and Fe1.5WO_*x*_ (Table S3); pseudo-first-order kinetic constants for the inhibition reactions using the Fe0.1WO_*x*_ photocatalyst (Table S4) (PDF)
