# Supplementary material for: Issues of Using Benzyl Ether in Nanomaterials’ Synthesis: Insights for a Standardized Synthesis of FeWO x Nanocrystals and Their Use as Photocatalysts
Source: ACS Omega. 2025 Sep 30;10(40):47609–22. doi: 10.1021/acsomega.5c07938 (PMC12529151; doi:10.1021/acsomega.5c07938)
Supplement: Supplementary file 1 [file ao5c07938_si_001.pdf]

SUPPORTING INFORMATION

Issues of Using Benzyl Ether in Nanomaterials Synthesis. Insights for a Standardized Synthesis of FeWOx Nanocrystals and Their Use as Photocatalysts

Raúl Boix[a,b], M. Pilar Lobera[a,b] and María Bernechea[a,b,c,]*

a Instituto de Nanociencia y Materiales de Aragón (INMA), CSIC-Universidad de Zaragoza. Department of Chemical and Environmental Engineering. C/ Mariano Esquillor s/n. 50018. Zaragoza. Spain.

b Centro de Investigación Biomédica en Red de Bioingeniería, Biomateriales y Nanomedicina, Instituto de Salud Carlos III, C/ Mariano Esquillor s/n. 50018. Zaragoza. Spain.

c ARAID, Government of Aragon, Av. Ranillas, 1-D. 50018. Zaragoza. Spain

* **mbernechea@unizar.es**

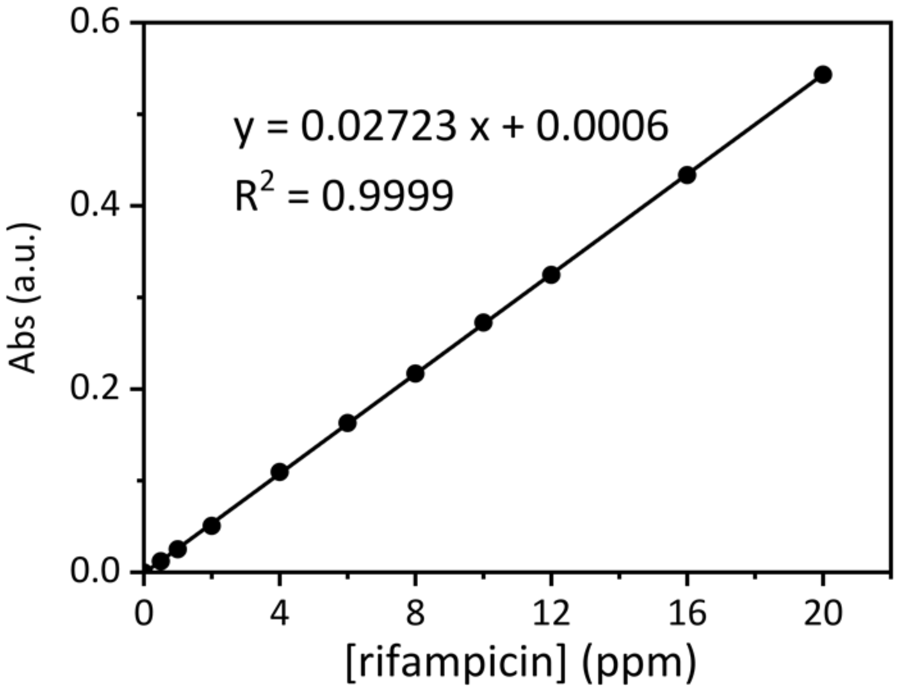

Figure S1. Calibration curve for rifampicin antibiotic according to absorbance values at 335 nm.

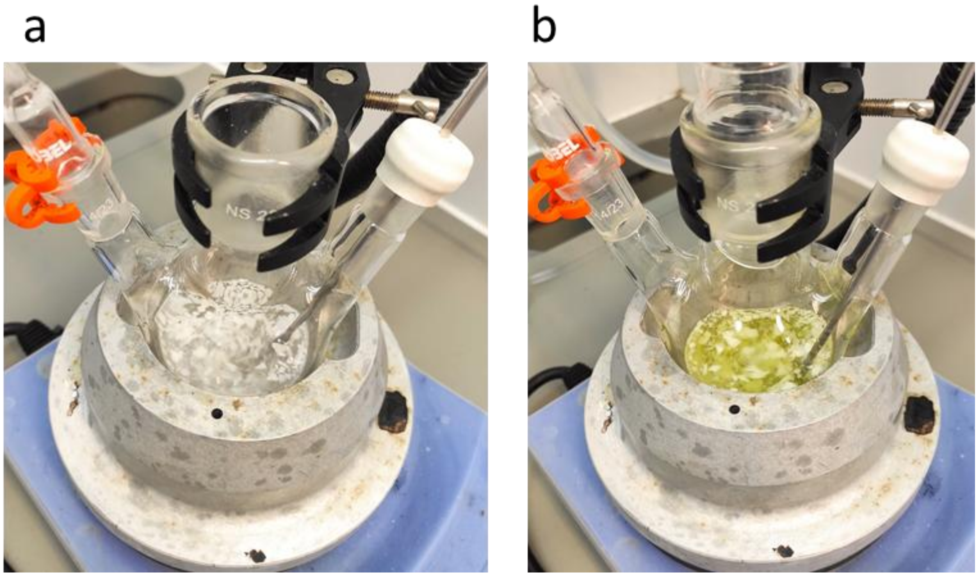

Figure S2. Colour changes observed in the reaction crude of $FeWO_x$ after the addition of 2.5 mmol oleylamine (a) colourless just before the addition; (b) yellow after the addition.

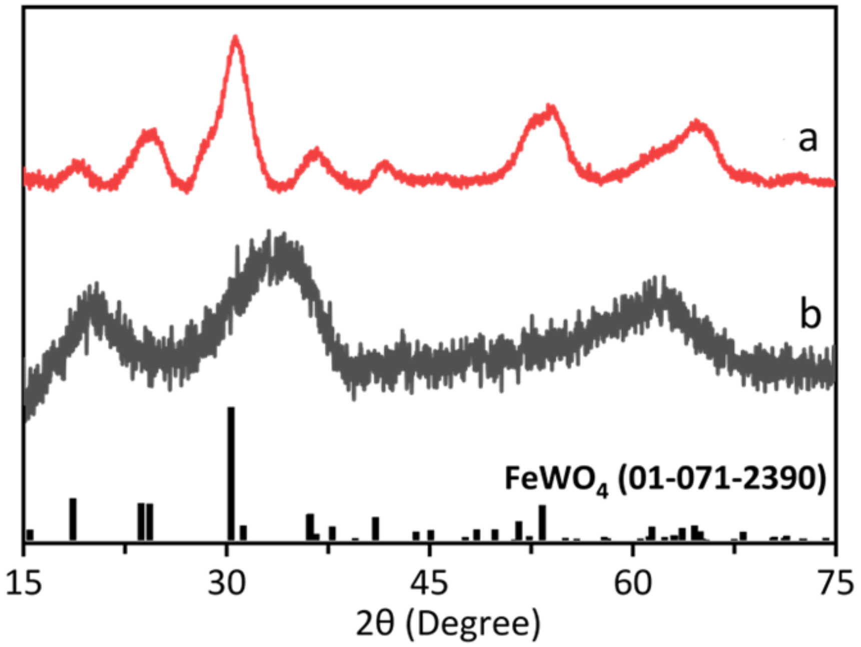

Figure S3. Representative XRD patterns of the materials obtained (a) before and (b) after the reproducibility problems of the synthesis ($Fe(acac)_3$ added at 170 ^{o}C, oleic acid (OA) and oleylamine (OAm) added at RT).

Table S1. Fe/W ratios, concentration of Fe (mM) and W (mM) determined by ICP-AES, mg of material obtained in each synthesis and % of organic content determined by TGA.

Compound[a)]	*Fe/W (MP – AES)*	*[Fe] (mM) (ICP-AES)*	*[W] (mM) (ICP-AES)*	*Amount obtained per synthesis (mg)*	*% Organic (wt./wt.) TGA*
FeWO_OA18	1.3 ± 0.04	0.052 ± 0.001	0.040 ± 0.004	92	24.3
FeWO_OA9	2.4 ± 0.03	0.061 ± 0.002	0.025 ± 0.003	85	41.5
FeWO_OA3_BA18	1.4 ± 0.01	0.059 ± 0.001	0.043 ± 0.001	140	19.0
FeWO_OA3_BA9	2.7 ± 0.02	0.069 ± 0.001	0.026 ± 0.003	105	39.5
FeWO_OA3_BA3	2.2 ± 0.01	0.055 ± 0.001	0.025 ± 0.002	85	40.9
FeWO_BA18	1.4 ± 0.02	0.058 ± 0.002	0.041 ± 0.001	90	24.8
FeWO_BA9	4.7 ± 0.01	0.056 ± 0.001	0.012 ± 0.001	126	54.5
FeWO_BA3	4.3 ± 0.02	0.064 ± 0.002	0.015 ± 0.002	108	48.5

a) The characterization of the FeWO_OA3 sample could not be carried out due to its high viscosity.

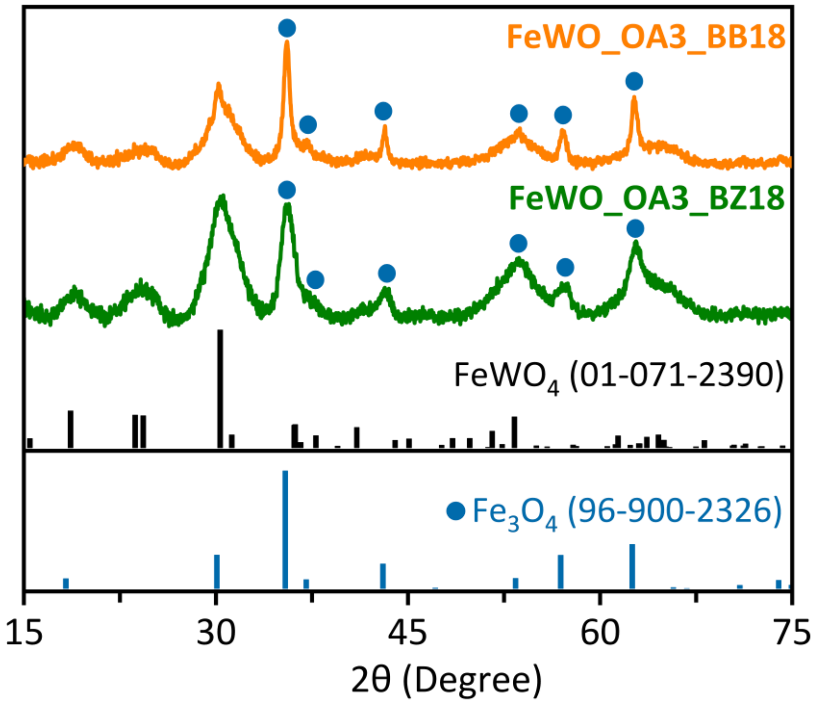

Figure S4. XRD pattern of the materials obtained adding 3 mmol of oleic acid (OA) and 18 mmol of benzyl benzoate (orange) or benzaldehyde (green) at RT, $Fe(acac)_3$ is added at 170 ^oC.

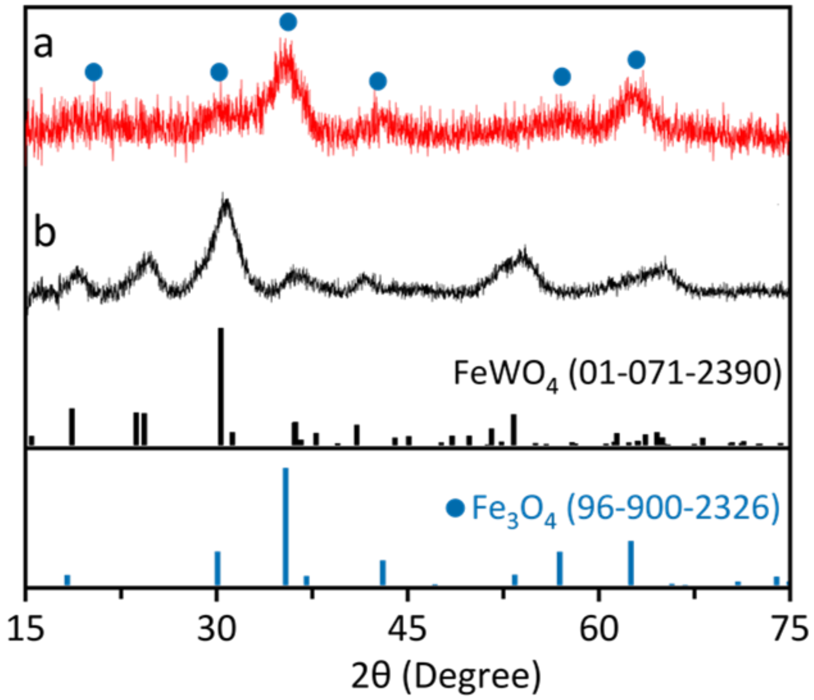

Figure S5. XRD patterns obtained using 1-octadecene as the solvent in FeWOx synthesis. With the incorporation of 1.5 g of 1,2-dodecanediol, 3 mmol of oleic acid (OA), and 2.5 mmol of OAm at room temperature, and the addition of $Fe(acac)_3$ at 170 ^oC; (a) without the addition of benzoic acid (BA), and (b) with the introduction of 18 mmol of benzoic acid (BA) at RT.

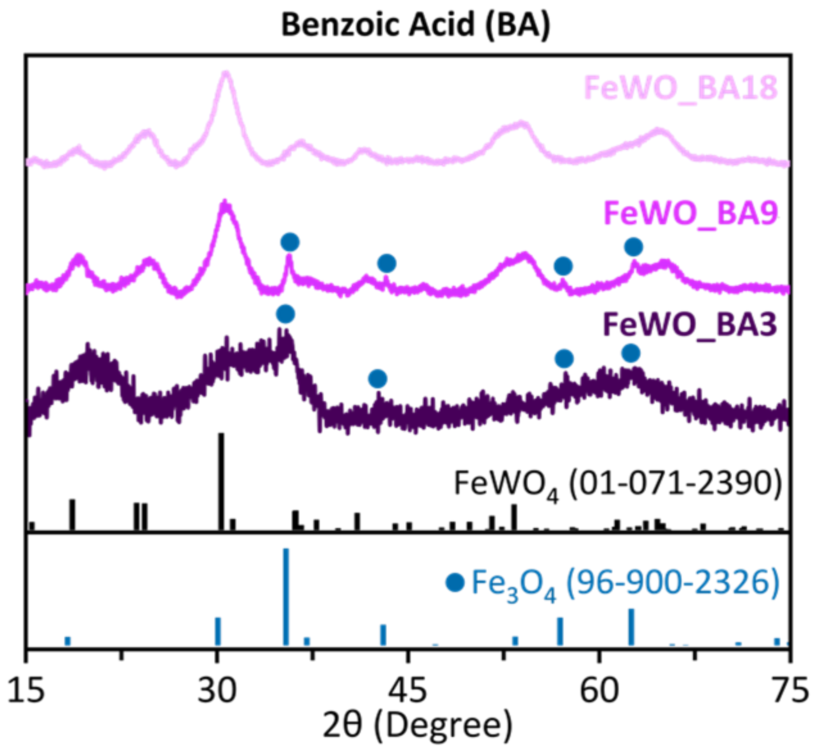

Figure S6. XRD patterns of the materials obtained without incorporating oleic acid (OA) and using different quantities of benzoic acid (BA) (3, 9 and 18 mmol) at RT, $Fe(acac)_3$ is incorporated at 170 ^{o}C.

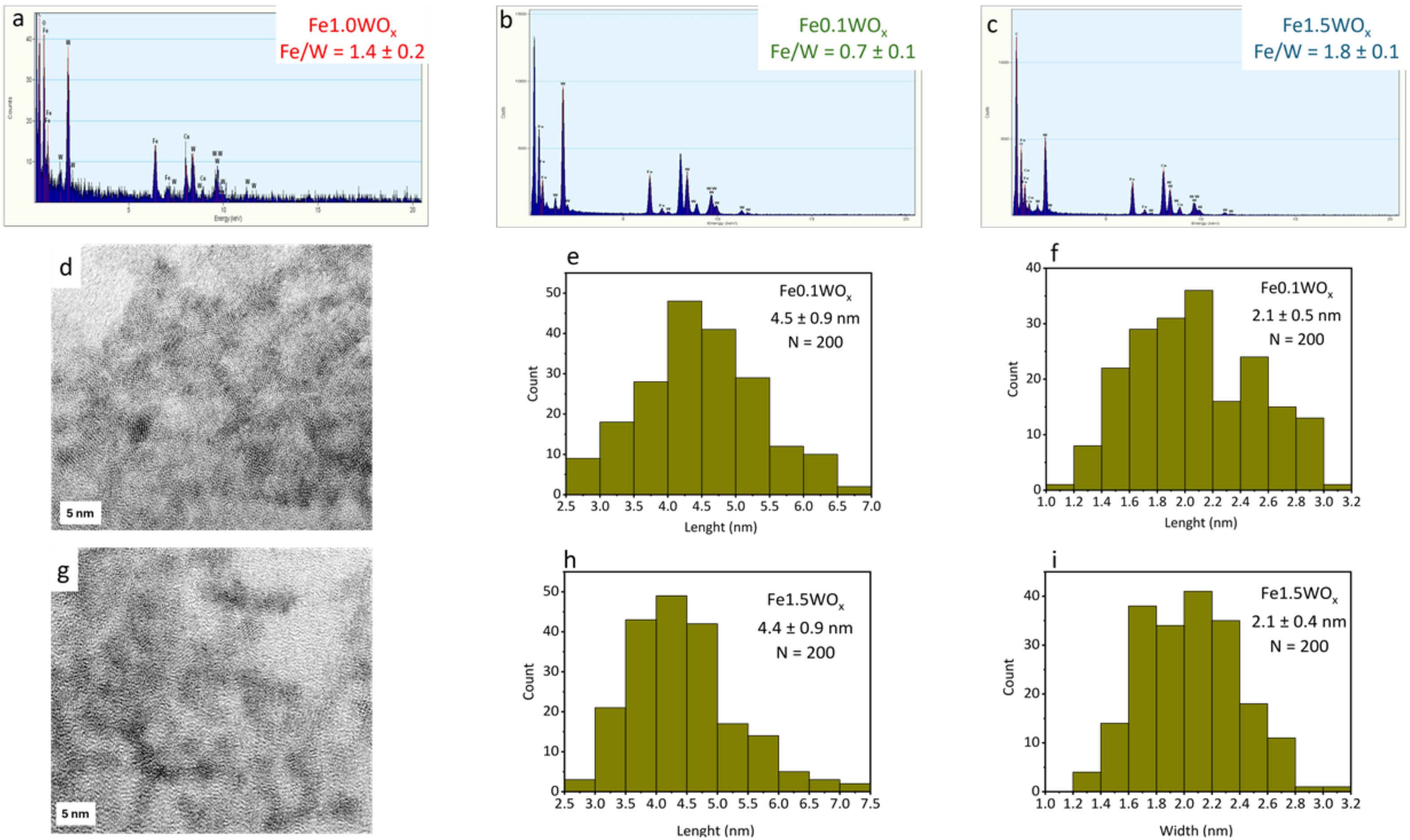

Figure S7. EDX-TEM spectra of (a) Fe1.0WO$_x$, (b) Fe0.1WO$_x$ and (c) Fe1.5WO$_x$. TEM images of (d) Fe0.1WO$_x$ and (g) Fe1.5WO$_x$. Length histograms of (e) Fe0.1WO$_x$ and (h) Fe1.5WO$_x$. Width histograms of (f) Fe0.1WO$_x$ and (i) Fe1.5WO$_x$.

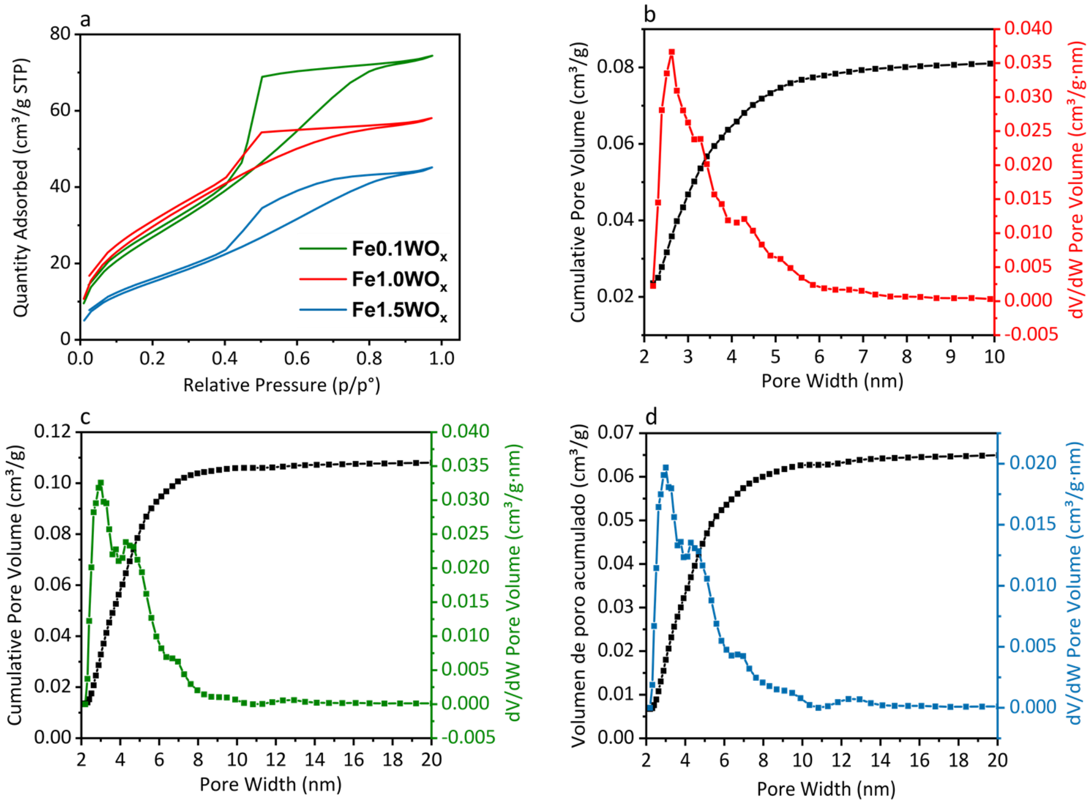

Figure S8. (a) BET nitrogen adsorption isotherm plot of Fe0.1WO$_x$ (green), Fe1.0WO$_x$ (red) and Fe1.5WO$_x$ (blue). DFT method - pore size and pore volume distribution of (b) Fe1.0WO$_x$ (red), (c) Fe0.1WO$_x$ (green) and (d) Fe1.5WO$_x$ (blue).

Table S2. Fe/W ratios, concentration of Fe (mM) and W (mM) determined by ICP-AES. Fe/W, Fe^{2+}/Fe^{3+}, W^{5+}/W^{6+} atomic ratios obtained by XPS. Fe/W atomic ratios quantified by EDX-SEM. Zeta potential, production (mg) of material obtained in each synthesis and % of organic content determined by TGA.

Compound	***ICP-AES***			***XPS***			***EDX***		***TGA***	***Zeta***	***Amount***
	Fe/W	***[Fe] (mM)***	***[W] (mM)***	***Fe/W***	***Fe^{2+}/Fe^{3+}***	***W^{5+}/W^{6+}***	***Fe/W (SEM)***	***Fe/W (TEM)***	***% Organic (wt./wt.)***	***potential (mV)***	***obtained per synthesis (mg)***
$Fe1.5WO_x$	1.6 ± 0.02	0.076 ± 0.001	0.049 ± 0.001	1.7 ± 0.1	1.2	1.1	1.7 ± 0.3	1.8 ± 0.1	17.0	-14.1 ± 0.7	69
$Fe1.0WO_x$	1.4 ± 0.01	0.072 ± 0.001	0.050 ± 0.001	1.2 ± 0.1	1.0	1.1	1.5 ± 0.3	1.4 ± 0.2	19.0	-24.9 ± 0.4	140
$Fe0.5WO_x$	1.1 ± 0.02	0.054 ± 0.001	0.047 ± 0.003	-	-	-	1.4 ± 0.3	-	17.2	-22.2 ± 0.5	112
$Fe0.3WO_x$	1.1 ± 0.01	0.053 ± 0.001	0.048 ± 0.006	-	-	-	1.3 ± 0.2	-	17.6	-25.8 ± 0.5	68
$Fe0.1WO_x$	1.1 ± 0.01	0.067 ± 0.001	0.058 ± 0.001	0.9 ± 0.1	1.9	1.0	1.3 ± 0.1	0.7 ± 0.1	19.0	-27.9 ± 0.8	43

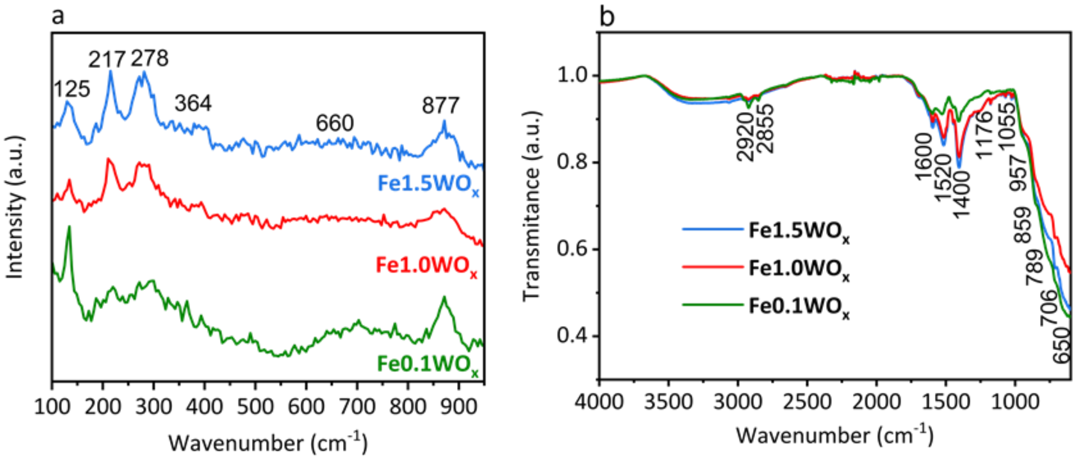

Figure S9. (a) Raman spectroscopy plot of $Fe0.1WO_x$ (green), $Fe1.0WO_x$ (red) and $Fe1.5WO_x$ (blue). (b) FTIR spectroscopy plot of $Fe0.1WO_x$ (green), $Fe1.0WO_x$ (red) and $Fe1.5WO_x$ (blue).

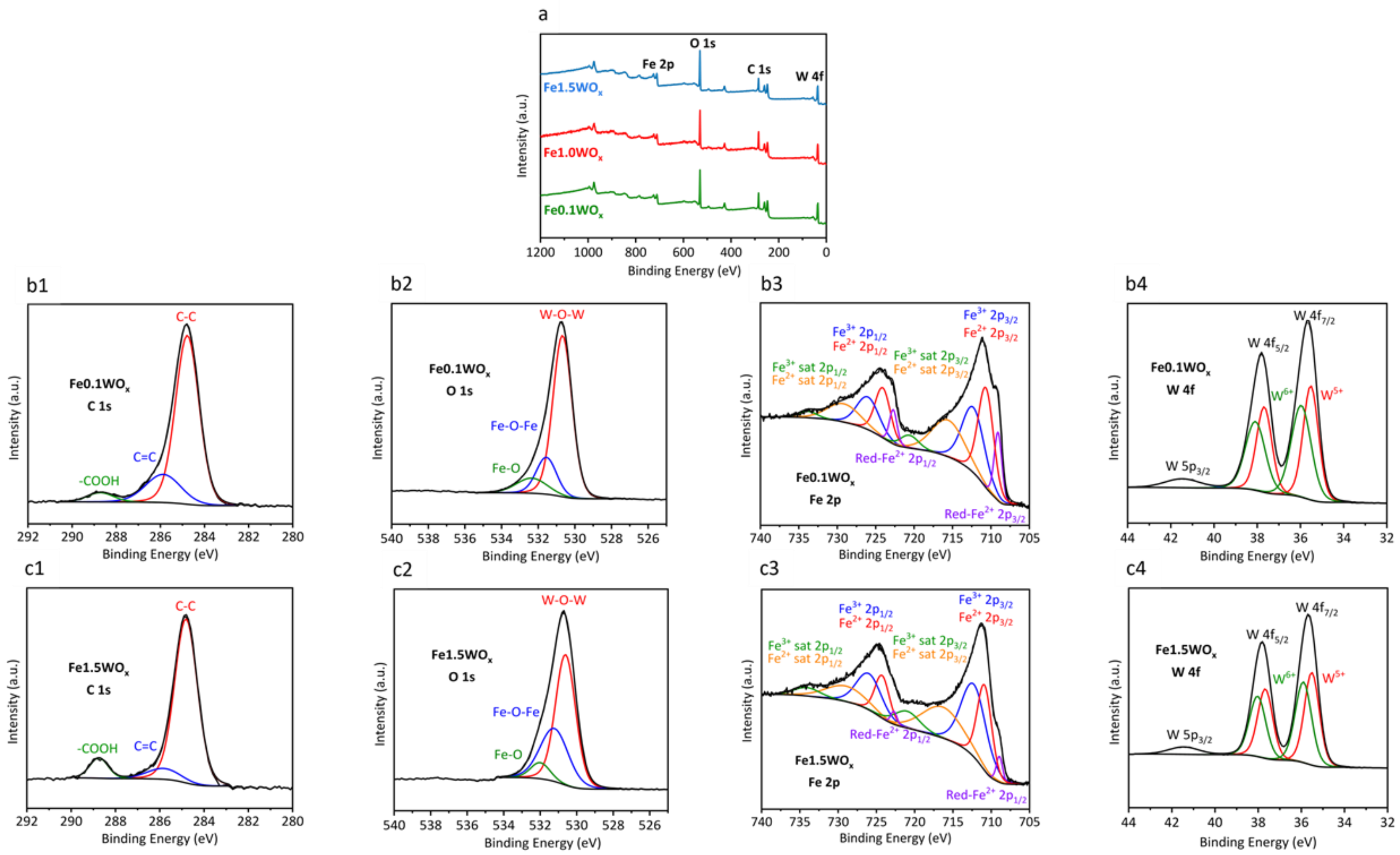

Figure S10. (a) General XPS spectra of Fe0.1WO_x, Fe1.0WO_x and Fe1.5WO_x materials. Detailed XPS spectra of (b1 and c1) C 1s, (b2 and c2) O 1s, (b3 and c3) Fe 2p and (b4 and c4) W 4f of Fe0.1WO_x and Fe1.5WO_x.

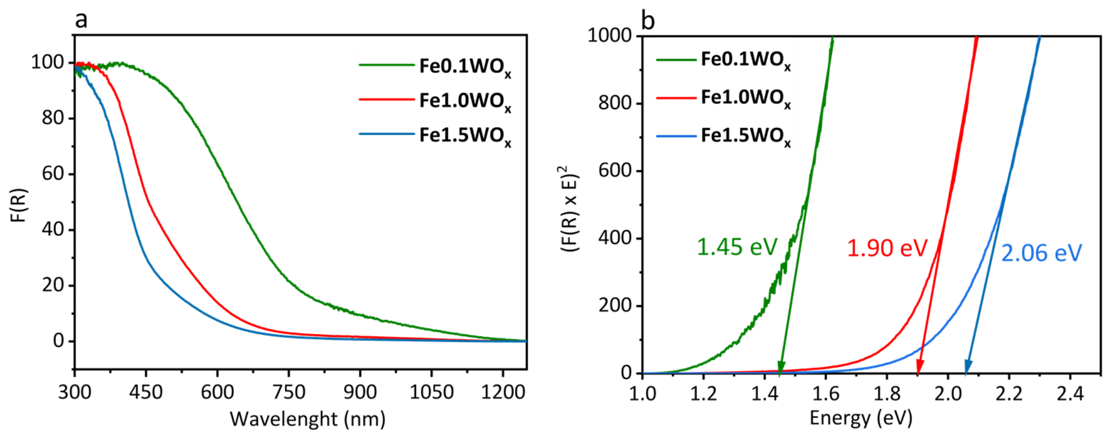

Figure S11. (a) UV-Vis DRS spectra (b) and Tauc plots of Fe0.1WO$_x$ (green), Fe1.0WO$_x$ (red) and Fe1.5WO$_x$ (blue) materials.

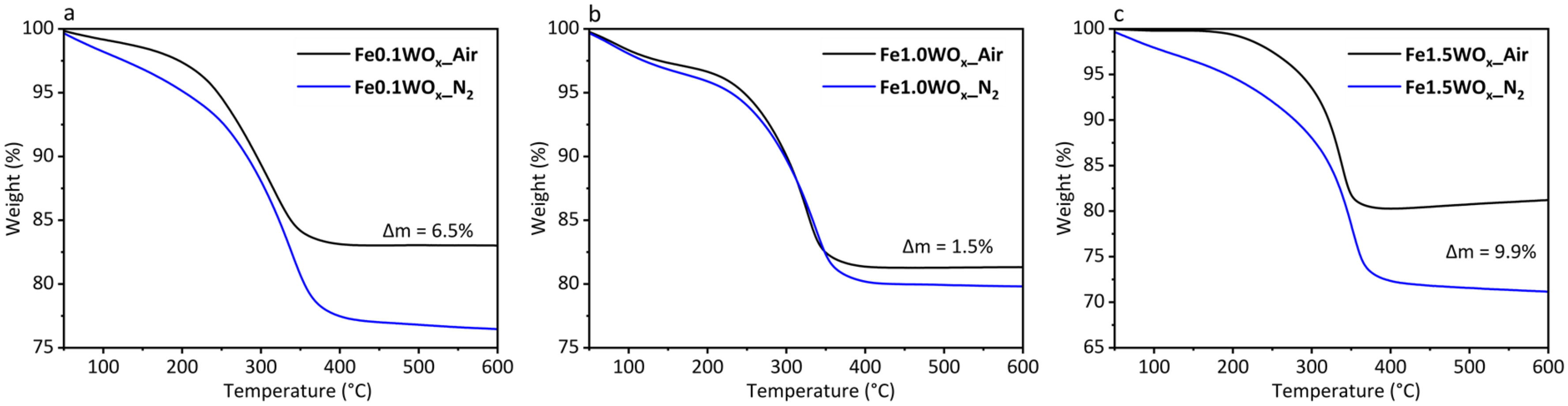

Figure S12. TGA analysis in air (black) and N_2 (blue) of (a) Fe0.1WO$_x$, Fe1.0WO$_x$ (b) and Fe1.5WO$_x$ (c). The measurements were made under 60 mL/min air or N_2 flow, with a heating ramp of 5 ^oC/min from 40 ^oC to 600 ^oC.

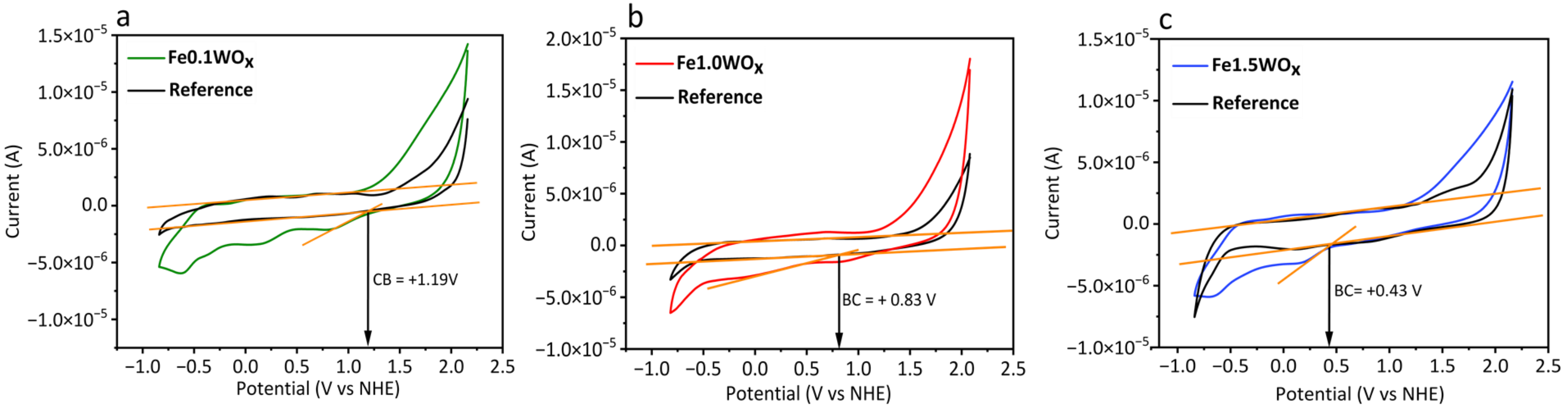

Figure S13. Cyclic voltammetries of (a) Fe0.1WO$_x$, (b) Fe1.0WO$_x$ and (c) Fe1.5WO$_x$: 50 mV/s in solutions of $TBAPF_6$ (0.2 M) in acetonitrile.

Table S3. Pseudo-first-order kinetic constants (k) of Fe0.1WO_x, Fe1.0WO_x, and Fe1.5WO_x.

Compound	*k (min^{-1})*	*R^2*
Photolysis	0.0014	0.9922
Fe1.5WO_x	0.0021	0.9961
Fe1.0WO_x	0.0023	0.9777
Fe0.1WO_x	0.0076	0.9939

Table S4. Pseudo-first-order kinetic constants (k) for the inhibition reactions using the Fe0.1WO_x photocatalyst.

Scavenger	*k (min^{-1})*	*R^2*
NaCl (h^+)	0.0023	0.9975
Isopropanol ($^{\bullet}OH$)	0.0047	0.9977
$NaNO_3$ (e^-)	0.0060	0.9976
Ascorbic Acid ($^{\bullet}O_2^-$)	0.0074	0.9918
